# Conserved spatial patterning of gene expression in independent lineages of C_4_ plants

**DOI:** 10.1111/nph.70475

**Published:** 2025-08-22

**Authors:** Tianshu Sun, Venkata Suresh Bonthala, Benjamin Stich, Leonie H. Luginbuehl, Julian M. Hibberd

**Affiliations:** ^1^ Department of Plant Sciences University of Cambridge Downing Street Cambridge CB2 3EA UK; ^2^ Julius Kuehn‐Institute, Federal Research Center on Cultivated Plants Institute for Breeding Research on Agricultural Crops 18190 Sanitz Germany

**Keywords:** C_4_ photosynthesis, evolution, *Flaveria bidentis*, *Gynandropsis gynandra*, single‐nucleus sequencing, transcription factors

## Abstract

C_4_ photosynthesis enhances carbon fixation efficiency by reducing photorespiration through the use of an oxygen‐insensitive carboxylase and spatial separation of photosynthesis between mesophyll and bundle sheath cells. The C_4_ pathway has evolved independently in > 60 plant lineages, but molecular mechanisms underpinning this convergence remain unclear. To explore this, we generated high‐resolution transcriptome atlases for two independently evolved C_4_ dicotyledonous species – *Gynandropsis gynandra* (NAD‐malic enzyme subtype) and *Flaveria bidentis* (NADP‐malic enzyme subtype). We used both single‐cell and single‐nucleus RNA sequencing to capture gene expression profiles from individual leaf cells, enabling a detailed comparison between cell types and transcriptional signatures. While both approaches produced biologically comparable data for major leaf cell types, transcriptomes from single‐nucleus sequencing showed lower stress signatures and were more representative of tissue proportions in the leaf. The single‐nucleus data revealed that bundle sheath cells from both C_4_ species share a gene expression pattern associated with mesophyll cells of C_3_ plants. A conserved set of transcription factors, including members of the C_2_H_2_ and DOF families, was identified in the bundle sheath cells of both species. This study presents the first single‐cell‐resolution transcriptomes for two independent C_4_ dicot lineages and provides a valuable resource, including a web‐based portal for data visualisation.

## Abstract 2

See also the Commentary on this article by Yee & Furbank, **249**: 5–7.

**Table:** 

	Contents	
	Summary	24
I.	Introduction	24
II.	Materials and Methods	26
III.	Results	27
IV.	Discussion	34
	Acknowledgements	35
	References	35

## I. Introduction

Photosynthesis sustains much of life on Earth. Central to the photosynthetic process is the enzyme ribulose‐1,5‐bisphosphate carboxylase‐oxygenase (RuBisCO), which fixes atmospheric carbon dioxide into the C_3_ compound 3‐phosphoglyceric acid. While RuBisCO predominantly catalyses this carboxylation reaction, it can also bind and react with oxygen to produce the toxic metabolite 2‐phosphoglycolate. The photorespiratory pathway allows the metabolism of 2‐phosphoglycolate, but requires additional energy input and leads to the loss of fixed nitrogen. Because the rate of oxygenation of RuBisCO increases with temperature, photorespiration becomes increasingly significant close to the equator (Schluter & Weber, 2020; Smith *et al*., 2023). To mitigate these effects, multiple lineages of plants have evolved carbon‐concentrating mechanisms that reduce the oxygenation reaction of RuBisCO, and thus rates of photorespiration. One such example is C_4_ photosynthesis (Hibberd & Covshoff, 2010; Hibberd, *et al*., 2008; Williams *et al*., 2012).

In contrast to the majority of plants where photosynthesis is primarily associated with mesophyll cells, in C_4_ species the process is typically equally divided between cell types, such as the mesophyll and bundle sheath. Thus, in C_4_ plants, carbon fixation operates across different cell types rather than within a single cell. Initially, HCO_3_^−^ is fixed in the mesophyll by phospho*enol*pyruvate carboxylase (PEPC) into C_4_ acids, which can diffuse to the bundle sheath. Subsequent decarboxylation of these C_4_ acids in the bundle sheath creates a CO_2_‐rich environment around RuBisCO. This compartmentation of the photosynthetic process enhances carbon fixation efficiency and reduces the need for photorespiration. However, the transition from C_3_ photosynthesis to the two‐celled C_4_ pathway requires significant optimisation of leaf anatomy, cell biology and biochemistry – all of which are underpinned by the rewiring of pre‐existing gene networks (Schluter & Weber, 2016). Despite this complexity, C_4_ photosynthesis has independently evolved > 60 times across multiple lineages of land plants (Sage *et al*., 2011). Although some C_4_ traits are underpinned by parallel evolution (Christin *et al*., 2007; Brown *et al*., 2011; Aubry *et al*., 2014), it has been known for some time that many characteristics of C_4_ photosynthesis are convergent (Christin & Osborne, 2013; Sage *et al*., 2012; Williams *et al*., 2013). This includes, for example, variation in Kranz anatomy as well as the biochemical basis of the pathway. For example, while in most cases bundle sheath cells have been co‐opted for the photosynthetic carbon reduction cycle, in some species use the mestome sheath for this purpose (Khoshravesh *et al*., 2020, 2016; Sage, 2004). Moreover, enzymes used to decarboxylate C_4_ acids during the photosynthetic carbon reduction cycle belong to three classes of protein – NADP‐malic enzyme (NADP‐ME), NAD‐malic enzyme (NAD‐ME) and PEP carboxykinase (PEPCK; Niklaus & Kelly, 2019; Schluter & Weber, 2020). The molecular basis of this convergent evolution of C_4_ traits, however, remains poorly understood (Westhoff & Gowik, 2010).

One reason for limited progress in understanding the molecular basis of C_4_ photosynthesis is associated with the complexity of separating specific cell types from leaves. Mechanical separation of cell types allowed mesophyll cells to be isolated from bundle sheath strands, but the latter are composed of xylem and phloem as well as the bundle sheath itself (Sheen, 1995; Covshoff *et al*., 2013). Protoplasting has also been used to isolate and study mesophyll and bundle sheath strands, but the process of isolating protoplasts induces stress responses to gene expression (Sawers *et al*., 2007). We reasoned that the advent of single‐cell and single‐nucleus sequencing technologies may provide tools to better understand the molecular basis of the convergent evolution of C_4_ photosynthesis. For example, droplet‐based single‐cell RNA‐seq technologies have significantly increased the throughput and resolution of transcriptome analyses (Cuperus, 2022; Chen *et al*., 2023). However, capturing the full diversity of cells in complex tissues using single‐protoplast RNA‐seq can be challenging due to the trade‐off between recovery of all cell types and longer incubation periods required for release of these cells causing stress responses in gene expression (Kim *et al*., 2021). In leaves, this issue can be exacerbated where vascular and bundle sheath cells are located deep in the tissue and digestion of cell walls is slow. Moreover, even after optimising protoplasting, some cell types can remain under‐represented, with, for example, < 2% bundle sheath cells being captured, and limited numbers of vascular or epidermal cells being retained (Bezrutczyk *et al*., 2021). In mammalian studies, single‐nucleus RNA‐seq has proved valuable for analysing tissues, such as neurons (Lacar *et al*., 2016), which are challenging to dissociate into single‐cell suspensions. It has also helped minimise gene expression changes that may arise from dissociation (van den Brink *et al*., 2017; Ding *et al*., 2020). Although transcript abundance is lower than in single‐cell RNA‐seq, the single‐nucleus method has demonstrated strong sensitivity and effectively classified cell types (Lake *et al*., 2017; Bakken *et al*., 2018; Guillotin *et al*., 2023). Moreover, recently single‐nucleus RNA‐seq has been used to assess gene expression during photomorphogenesis in C_3_ and C_4_ grasses (Swift *et al*., 2024).

We therefore set out to compare single‐cell and single‐nucleus RNA sequencing from two species that evolved C_4_ photosynthesis independently. Specifically, we chose *Gynandropsis gynandra* and *Flaveria bidentis* that belong to the dicotyledons, have previously been used to study C_4_ photosynthesis and have annotated genome sequences. *Gynandropsis gynandra* (formerly Cleome) uses NAD‐ME as the primary decarboxylase in bundle sheath cells (Brown *et al*., 2005; Marshall *et al*., 2007), is a leafy vegetable widely grown across Sub‐Saharan Africa and South East Asia (Mashamaite *et al*., 2022) and is sister to the Brassicaceae and therefore closely related to *Arabidopsis thaliana*. Comparative analysis between *G. gynandra* and *A. thaliana* has provided insight into multiple aspects of the complex C_4_ phenotype, including the formation of plasmodesmata (Schreier *et al*., 2024), evolution of the genome (Hoang *et al*., 2023) and the rewiring of gene networks responsive to light, as well as associated with cell‐specific gene expression and cell identity (Singh *et al*., 2023). *Flaveria bidentis* uses NADP‐ME as the primary C_4_ acid decarboxylase (Gowik *et al*., 2011), and comparative analysis of species in the genus formed the foundations for many of the conceptual and mathematical models that are used to explain why and how C_4_ photosynthesis evolved (Heckmann *et al*., 2013; Sage *et al*., 2013; Schulze *et al*., 2013). *G. gynandra* and *F. bidentis* belong to the rosid and asterid angiosperm clades, the two largest eudicot clades, which diverged at least 100 million years ago (Ma) (Moore *et al*., 2010; Zuntini *et al*., 2024).

We compiled single‐protoplast and single‐nucleus transcriptome atlases for all major cell types of *G. gynandra* and *F. bidentis* leaves, and provide a browser‐based interactive tool to allow exploration of the data (at https://hibberdlab.shinyapps.io/gg_integrated/ for *G. gynandra* and https://hibberdlab.shinyapps.io/fb_integrated/ for *F. bidentis*). Both protoplast and nucleus sequencing produced biologically meaningful and comparable cell‐type‐specific data for the major cell types in a C_4_ leaf, but single‐nucleus sequencing provided more accurate cell type representation, and transcriptomes lacked protoplast‐induced stress responses. Analysis of transcript abundance in all major cell types of leaves allowed us to investigate the extent to which mesophyll and bundle sheath cells from these independent C_4_ lineages shared the same molecular signatures. Despite representing distinct C_4_ lineages and using different decarboxylation enzymes in the bundle sheath, in both *G. gynandra* and *F. bidentis* compared with the ancestral C_3_ state, bundle sheath cells gained expression of a similar set of genes, previously associated with mesophyll cells and allowing decarboxylation and carbon fixation in the C_4_ cycle. Furthermore, we identify transcription factor families, such as IDD and DOFS, that are preferentially expressed in either mesophyll or bundle sheath cells of both species.

## II. Materials and Methods

### 1. Plant growth conditions

*Gynandropsis gynandra* (L.) seeds were germinated on moist filter paper in the dark at 32°C for 32 h and then sown directly onto soil. *Flaveria bidentis* (L.) seeds were placed on ½‐strength Murashige and Skoog medium in 0.8% (w/v) agar (pH 5.8) and grown for 10 d in a growth chamber at 24°C, 16 h : 8 h, light : dark photoperiod before being transferred to soil. After sowing *G. gynandra* and transplanting *F. bidentis*, plants were maintained in a growth cabinet under a 16 h : 8 h, light : dark cycle at 24°C, with 60% relative humidity and a photon flux density of 400 μmol m^−2^ s^−1^. Plants were grown for 6 wk until leaves were harvested for single‐protoplast or single‐nucleus RNA sequencing. To generate DNA for genome assembly, *F. bidentis* seeds were germinated and grown in growth chambers at Heinrich Heine University, Dusseldorf, Germany. Samples from multiple organs – leaves, stem and root – were collected and frozen in liquid nitrogen. Genomic DNA was extracted from leaves and RNA from all organs above‐mentioned using the Qiagen DNEasy Mini Kit following the manufacturer's protocol (Qiagen, Germany).

### 2. Whole‐genome sequencing, estimation of genome size, reference‐quality genome assembly and genome annotation

The genome of *F. bidentis* was sequenced at NRGene, Nes Ziona, Israel using 10X linked‐read and Illumina sequencing technologies. Genome size was estimated using the K‐mer (127 bp) coverage distribution, and the heterozygous (Het) and homozygous (Homo) peaks of unique K‐mer distributions were chosen for genome size estimation. The estimated genome size was then calculated by the division of all K‐mer by the coverage of unique K‐mers of Het peaks.

The 10× linked‐read and Illumina sequence libraries were used for *de novo* genome assembly using the DeNovoMAGIC pipeline (NRGene, Nes Ziona, Israel), a DeBruijn‐graph‐based genome assembler to generate a reference‐quality assembly. We assessed the contiguity of the assembly using the N50 metric. The completeness of the assembly was assessed by evaluating the presence of conserved single‐copy orthologous genes using Benchmarking Universal Single‐Copy Orthologs (BUSCO) v.5.5.0 against the ‘embryophyta_odb10’ lineage dataset (Seppey *et al*., 2019).

We performed gene annotation using Helixer v.0.3 (Stiehler *et al*., 2021), a deep learning‐based gene annotation tool with a training dataset of ‘land_plant’ to annotate protein‐coding genes. Furthermore, we performed functional annotation for predicted genes using the standalone version of InterProScan v.5.57–90.0 (Jones *et al*., 2014). In addition, gene descriptions were assigned by performing blastp (Altschul *et al*., 1990) with default parameters against the UniProt protein database (downloaded on 29 August 2022 from https://www.uniprot.org). Finally, gene models were removed that produced protein sequences with < 50 amino acids and gene models related to transposable elements using a curated list of Pfam protein domain IDs (Jayakodi *et al*., 2020).

We combined the extracted RNA from multiple organs into a single library and sequenced it using Oxford Nanopore direct RNA sequencing (DRS) technology at the Genomics & Transcriptomics Lab, Heinrich Heine University, Düsseldorf, Germany. The DRS reads were subjected to quality filtering using NanoFilt v.2.8.0 (De Coster *et al*., 2018) to remove reads with a length < 150 bases and quality *q* < 10, followed by trimming adapters using Porechop v.0.2.4 (Wick *et al*., 2017). Finally, the high‐quality reads were mapped against the reference genome, and expression abundance (TPM) was estimated using NanoCount v.1 (Gleeson *et al*., 2022). Genes with TPM (Transcripts Per Million) > 0 were considered as expressed.

### 3. Protoplast and nuclei isolation

For protoplast isolation, leaves were harvested and finely chopped in a 9 cm Petri dish containing 15 ml of freshly prepared enzyme cocktail (1.25% (w/v) cellulase RS, 0.3% (w/v) macerozyme R10, 0.2–0.6 M mannitol, 20 mM KCl, 20 mM MES, pH 5.7, 10 mM CaCl₂ and 0.1% (w/v) bovine serum albumin (BSA)). Tissue was incubated for 2.5 h on a shaking incubator (40 rpm, 25°C, dark), and digestion stopped by adding an equal volume of W5 buffer (2 mM MES, pH 5.7, 125 mM CaCl₂, 154 mM NaCl and 5 mM KCl). The released protoplasts were filtered twice using a 40‐μm cell strainer and then collected in a 50‐ml tube. Protoplasts were centrifuged at 100 ***g*** for 5 min and resuspended three times – twice in W5 buffer and then 0.4 M mannitol buffer for purification. After the final wash, the supernatant was gently removed, leaving 0.5–1 ml of protoplast suspension in 0.4 M mannitol. Protoplast integrity and concentration were assessed using a C‐Chip haemocytometer under a light microscope. Protoplast suspensions of good quality (> 80% integrity, minimal debris) were adjusted to a concentration of *c*. 1000 protoplasts μl^−1^.

Nuclei were isolated according to the published protocol (Swift *et al*., 2024). Briefly, leaves were harvested at the same development stage and finely chopped in a 5‐cm Petri dish containing 1.5 ml of freshly prepared nuclei purification buffer (10 mM Tris–HCl, pH 7.4, 10 mM NaCl, 3 mM MgCl₂, 0.5 mM spermidine, 0.2 mM spermine, 1× Roche Complete protease inhibitors, 0.2% (w/v) BSA and 1 U μl^−1^ Protector RNase Inhibitor). Tissue was filtered once through a 70‐μm and then through a 40‐μm cell strainer. Nuclei were stained with 0.5 μl Hoechst solution (Hoechst 33342, trihydrochloride, trihydrate; 10 mg ml^−1^ solution in water, Invitrogen) and sorted using a BD FACSAria™ III Cell Sorter with a 70‐μm nozzle. Sorted nuclei were collected in a 1.5‐ml tube containing BSA and Protector RNase Inhibitor and then counted using a C‐Chip haemocytometer under a light microscope. Nuclei suspensions were adjusted to a concentration of *c*. 400–600 nuclei μl^−1^.

### 4. Library preparation and sequencing, data processing and quality control

Single‐protoplast RNA‐seq of *F. bidentis* was performed with three biological replicates, while two biological replicates were carried out for all other samples. For each replicate, 10 000 protoplasts (targeting recovery of 6000 cells) or 16 000 nuclei (targeting recovery of 10 000 nuclei) were loaded per well according to the Chromium Next GEM Single Cell 3′ Reagent Kits v.3.1 protocol. cDNA for each sample was barcoded and amplified, and single‐cell libraries were constructed (dual index) and then sequenced using Illumina NovaSeq systems following the manufacturer's standard protocol.

Demultiplexed sequences were analysed using CellRanger v.7.0.0 (10× Genomics) with default parameters to generate initial gene–cell matrices (using intron mode). All samples were processed with default settings, except for the nuclei libraries from *G. gynandra*, which were analysed with ‐‐force‐cells = 10 000. Reads from *G. gynandra* were mapped to its genome assembly (v.3.0; Hoang *et al*., 2023), and reads from *F. bidentis* were mapped to the reference‐quality genome assembly that we generated in this study. To account for ambient RNA contamination, count matrices were adjusted using the adjustCounts() function from SoupX (Young & Behjati, 2020). DoubletFinder (McGinnis *et al*., 2019) was used to assess the probability of droplets containing more than one nucleus or protoplast, and predicted doublets were excluded from downstream analysis. Further quality control was performed using Seurat v.4.3.0 (Hao *et al*., 2021). Genes expressed in fewer than three cells, and cells with fewer than 200–500 genes (depending on dataset quality) were excluded from further analyses.

### 5. Clustering, cell type annotation, homologue identification, Gene Ontology and orthology analysis

Nuclei or protoplast libraries from the same species were merged using Seurat v.4.3.0 and batch‐corrected with harmony v.0.1.1 (Korsunsky *et al*., 2019). For the integration of nuclei and protoplast libraries, Seurat's FindIntegrationAnchors() function was used to identify anchor genes, followed by integration using IntegrateData(). Neighbour graph construction, cluster determination and nonlinear dimensionality reduction were performed with Seurat using FindNeighbors(), FindClusters() and RunUMAP() functions.

Homologues between *Arabidopsis thaliana, G. gynandra* and *F. bidentis* were identified using blastp v.2.12.0 (Camacho *et al*., 2009). Nuclei or protoplast clusters were annotated based on the expression of homologous genes corresponding to known leaf cell type markers from *Arabidopsis*. Clusters without clear marker gene expression or showing mixed expression patterns were labelled as ‘ua’ (unannotated). Annotation information was added to nuclei and protoplasts in the Shiny‐based web application for atlas visualisation, which was created using ShinyCell v.2.1.0 (Ouyang *et al*., 2021). Marker genes for each cell type were identified using Seurat's FindAllMarkers() function by comparing nuclei/protoplasts from a specific cell type against all other cell types. Differentially expressed genes (DEGs) between nuclei and protoplasts, or between two different cell types, were identified using FindMarkers(). Marker genes and DEGs were only considered if they showed a log fold change of at least 0.25 and were detected in a minimum fraction of 10% of cells in either population. The best BLAST hit against *Arabidopsis* of each marker gene/DEG was used for Gene Ontology (GO) enrichment analyses, and performed using clusterProfiler v.4.2.2 (Wu *et al*., 2021), with a false discovery rate cut‐off of 0.05. Stress and nuclei signature scores were calculated with the AddModuleScore() function in Seurat using the expression of genes enriched in respective pathways.

Compartmentation of orthologous genes across cell types in *A. thaliana, G. gynandra* and *F. bidentis* was based on orthogroups identified by OrthoFinder v.2.4 (Emms & Kelly, 2019), using protein sequences from primary transcripts of species, including *Zea mays* (RefGen_V4), *Oryza sativa* (v7.0), *Brachypodium distachyon* (v3.2), *Sorghum bicolor* (v3.1), *Vitis vinifera* (v2.1), *Theobroma cacao* (v2.1), *Arabidopsis thaliana* (Araport11), *Carica papaya* (ASGPBv0.4), *Malus domestica* (v1.1), *Fragaria vesca* (v4.0.a2), *Ricinus communis* (v0.1), *Manihot esculenta* (v8.1), *Populus trichocarpa* (v4.1), *Medicago truncatula* (Mt4.0v1), *Glycine max* (Wm82.a6.v1), *Solanum lycopersicum* (ITAG5.0), and *G. gynandra* and *F. bidentis*. The *Arabidopsis* single‐protoplast expression matrix (Kim *et al*., 2021) was used to compare cell type expression patterns between C_3_ and C_4_ plants. Nitrogen assimilation and amino acid metabolism genes were identified using Mercator4 (Bolger *et al*., 2021). Transcription factor families were classified based on the PlantTFDB (Jin *et al*., 2017). The co‐expression network was constructed and visualised with hdWGCNA (Morabito *et al*., 2023).

## III. Results

### 1. Optimised protocols for single‐nucleus and ‐protoplast isolation from *G. gynandra* and *F. bidentis*

To allow a direct comparison between single‐nucleus and single‐protoplast RNA‐seq from leaves of C_4_ plants, we optimised experimental protocols to obtain high‐quality suspensions of nuclei and protoplasts from *G. gynandra* and *F. bidentis* leaves (Supporting Information Fig. S1). Nuclei were isolated after chopping leaves, and individual nuclei were then purified using fluorescence‐activated cell sorting. To ensure clear separation of nuclei from debris and chloroplasts, we adjusted the gating for positive events by excluding those with high autofluorescence (Fig. S2). Purified nuclei were then used for library preparation. For protoplast isolation, enzyme composition, mannitol concentration and age of plants were assessed. Under our conditions, age had a major effect on protoplast viability – with those from older plants being less likely to burst (Fig. S3a,b). We also found that avoiding the commonly used protoplast purification solutions, which contain Na^+^, K^+^ and Ca^2+^, was crucial for efficient transcriptome capture, as their presence ultimately resulted in decreased gene detection (Fig. S3c–f).

With these optimised samples, we generated libraries using the 10× Chromium platform from nuclei and protoplasts of *G. gynandra* and *F. bidentis* and subjected these to sequencing. Reads from *G. gynandra* were mapped to the published genome (Hoang *et al*., 2023), while reads from *F. bidentis* were mapped to a reference‐quality genome we assembled for *F. bidentis* with N50 of 41.8 Mb, capturing 96.29% of complete BUSCO genes of the embryophytes (Table S1; Seppey *et al*., 2019). To generate this genome, we sequenced *c*. 884 Gb that represented a coverage of *c*. ×635 (Table S2). The genome size of *F. bidentis* was estimated as 1.39 Gb with the K‐mer coverage distribution approach, and this led to a predicted greater size (1.01 Gb) than that previously reported from flow cytometry (Taniguchi *et al*., 2021). Genome annotation produced 26 603 gene models with an average length of 3692.35 bases, and functional annotation assigned protein domain descriptions (PFAM) and GO terms to 80.4% and 52.79% of genes, respectively.

**Fig. 1:**
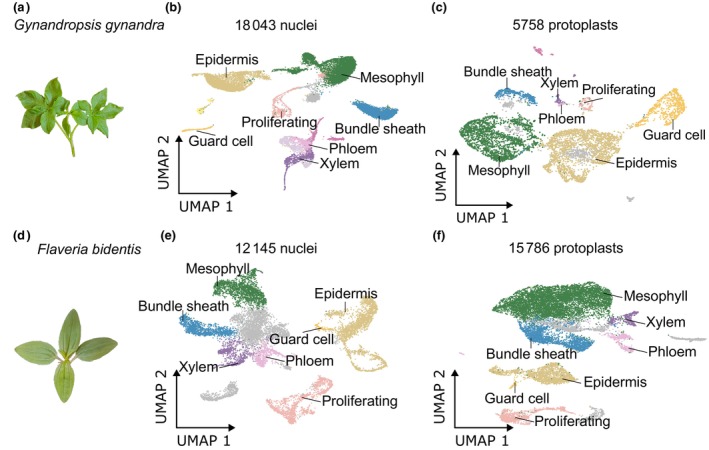
Single‐nucleus and single‐protoplast RNA sequencing from leaves of *Gynandropsis gynandra* and *Flaveria bidenti*s. (a, d) Leaves of *G. gynandra* and *F. bidentis* were sampled for the isolation of single nuclei and protoplasts for sequencing. Uniform Manifold Approximation and Projection (UMAP) visualisation of single‐nucleus (b, e) and single‐protoplast sequencing (c, f) from *G. gynandra* (b, c) and *F. bidentis* (d, e). Clusters are coloured by cell identity; unannotated cells (ua) are shown in grey.

After mapping raw reads to the respective genomes and performing doublet removal, replicate combination, quality control and batch effect removal, we generated gene expression matrices for all four datasets. For *G. gynandra*, we identified 22 224 genes from 18 043 nuclei (913 genes per nucleus) and 23 522 genes from 5 758 protoplasts (3 974 genes per protoplast; Fig. 1a–c). For *F. bidentis*, we identified 22 398 genes from 12 145 nuclei (548 genes per nucleus) and 23 979 genes from 15 786 protoplasts (1 945 genes per protoplast; Fig. 1d–f). The Uniform Manifold Approximation and Projection (UMAP) algorithm was used to visualise local similarities and global structures of each dataset, and identified up to 19 clusters in the single‐nucleus datasets and 15 clusters in the protoplast datasets (Fig. S4a–d). Transcriptome profiles were highly correlated between all replicates (Fig. S4e–h). Based on the expression of marker genes for leaf cell types, we identified mesophyll, bundle sheath, xylem, phloem, epidermis, guard and proliferating cell clusters in both single‐nucleus and single‐protoplast datasets (Figs 1, S4i–l). We conclude that both methods captured sufficient numbers of cells, allowed identification of major cell types and the quantification of gene expression. As expected, fewer genes were detected in nuclei compared with protoplasts.

### 2. Leaf cell atlases for two distinct lineages of C_4_ plant

**Fig. 2:**
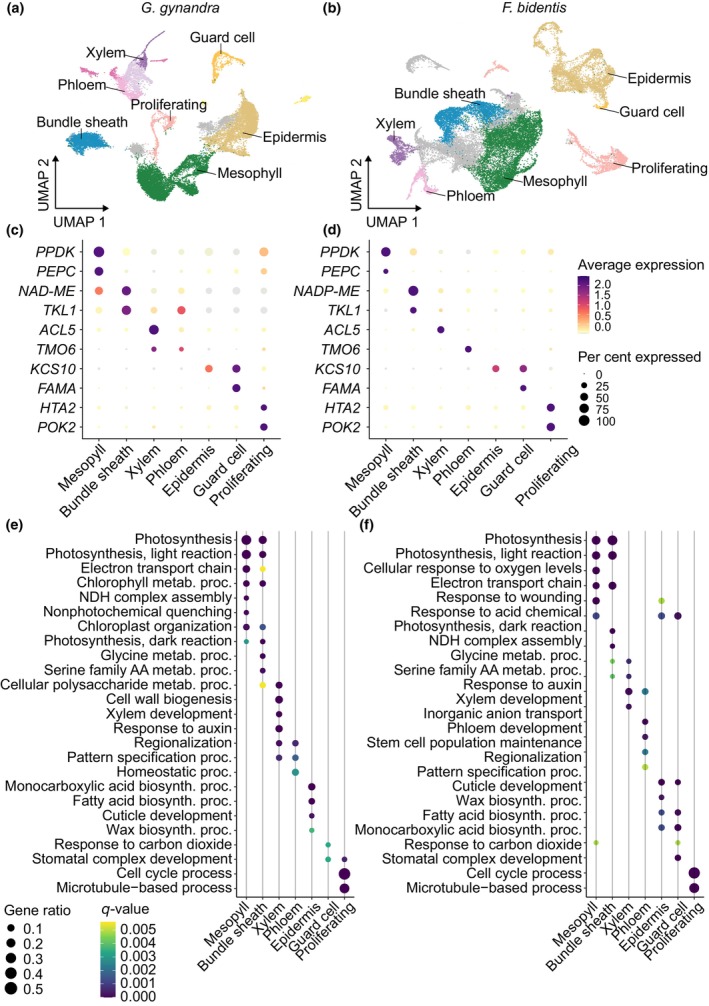
Integrated single‐nucleus and single‐protoplast atlases for *Gynandropsis gynandra* and *Flaveria bidenti*s leaves. (a, b) Uniform Manifold Approximation and Projection (UMAP) visualisation after integration of nucleus and protoplast data from *G. gynandra* (a) and *F. bidentis* (b), unannotated cells (ua) are shown in grey. (c, d) Dot plots showing the expression of marker genes for major cell types of *G. gynandra* (c) and *F. bidentis* (d) leaves. Dot size represents the percentage of nuclei and protoplasts in each cluster expressing the gene indicated. Colour indicates the average expression level, calculated as the mean expression of the gene across all nuclei and protoplasts in which it is detected in that cluster. (e, f) Dot plots displaying enriched Gene Ontology (GO) terms derived from the marker genes of each annotated cell type cluster for *G. gynandra* (e) and *F. bidentis* (f). Dot size represents the fraction of marker genes associated with each GO term relative to the total number of input marker genes, and colour corresponds to *q*‐value of enrichment. biosynth., biosynthetic; metb., metabolic; proc., process.

We integrated the single‐nucleus and single‐protoplast datasets and re‐clustered the combined dataset. This increased statistical power because more protoplasts/nuclei were available for each species. In *G. gynandra*, we detected the expression of 24 035 genes from 23 801 nuclei and protoplasts and identified a total of 18 clusters. For *F. bidentis*, 24 874 genes were detected from 27 931 nuclei and protoplasts, and these could be separated into 21 clusters (Fig. S5a,b). Each cluster was annotated based on the differential expression of previously defined marker genes (Figs 2a–d, S5c,d; Table S3). In *G. gynandra*, we identified all major leaf cell types, including mesophyll, bundle sheath, epidermis, guard cells, proliferating cells and vasculature cells (Fig. 2a,c). Mesophyll cells from *G. gynandra* (Clusters 0, 5 and 8) showed elevated expression of key genes required for the C_4_ cycle, such as *PEPC* and *PYRUVATE, ORTHOPHOSPHATE DIKINASE* (*PPDK*). Bundle sheath cells (Cluster 2) were identified based on strong expression of genes encoding the C_4_ acid decarboxylase NAD‐dependent malic enzyme (ME) and transketolase from the Calvin–Benson–Bassham cycle. Expression of the epidermis‐specific gene *3‐KETOACYL‐COA SYNTHASE 10* was predominantly detected in Clusters 1, 4, 16 and 7. Cluster 7 not only exhibited high expression of epidermis marker genes but also showed enriched expression of guard cell marker genes, such as *FAMA* (*FMA*). In previous studies, a histone H2A protein *HTA2* and *PHRAGMOPLAST ORIENTING KINESIN 2* (*POK2*) have been associated with cell proliferation during the cell cycle, especially during the synthesis phase and cytokinesis. In our dataset, we identified their expression mainly in Clusters 10 and 15, indicating that these two clusters likely represent proliferating cells. Expression of the xylem‐specific gene *ACAULIS5* (*ACL5*) was enriched in Clusters 3 and 14, while Cluster 3 also contained the phloem marker *TMO6*, suggesting that Cluster 3 represents a mixed population of vascular cell types. Subsetting and re‐clustering cells from both Cluster 3 and 14 allowed categorisation of phloem, xylem and procambial cell types (Fig. S5a,c). We used similar marker genes (the only difference was the use of *NADP‐ME* as a bundle sheath marker) to annotate cell types in *F. bidentis*, and this allowed us to annotate mesophyll (Clusters 1, 2, 5 and 19), bundle sheath (Clusters 3 and 8), xylem (Cluster 9), phloem (Cluster 15), epidermis (Clusters 4, 7, 12 and 17), guard cells (Cluster 18) and proliferating cells (Clusters 6 and 14; Figs 2b,d, S5b,d).

To gain insights into the functional specialisation of each cell type, we further analysed DEGs between all cell types (i.e. between one cell type and all others; *P* < 0.01; average log fold change ≥ 0.25; expressed in > 10% of cells with a specific cell type; Tables S4, S5). DEGs with strong specificity (Fig. S6) included both the marker genes we had initially used to annotate cell types, but also other genes that were highly specific. For example, in *G. gynandra*, we identified 1027 and 1345 marker genes for the mesophyll and bundle sheath, respectively, while in *F. bidentis* we found 2209 and 1257 for the mesophyll and bundle sheath. In both species, mesophyll markers included well‐known genes associated with the C_4_ cycle, such as *PPDK*, *PEPC* and photosystem subunits, while bundle sheath markers included genes required for the Calvin–Benson–Bassham cycle, such as *RIBULOSE BISPHOSPHATE CARBOXYLASE OXYGENASE SMALL SUBUNIT (RbcS*), *FRUCTOSE‐BISPHOSPHATE ALDOLASE1* (*FBA1*) or photorespiration, including *GLYCINE DECARBOXYLASE T‐SUBUNIT (GLD‐T*) and *SERINE HYDROXYMETHYLTRANSFERASE 1 (SHM1*; Tables S6, S7). We further performed GO enrichment analysis using the top 200 DEGs of each cell type. In both species, the top 200 DEGs from both mesophyll and bundle sheath cells were significantly enriched in GO terms annotated as photosynthesis, light reactions and electron transport, while those genes in bundle sheath cells were also enriched in terms associated with glycine metabolism and serine catabolism. Enrichment of marker genes for other cell types (xylem, phloem, epidermis, guard and proliferating cells) aligned with known functions (Fig. 2e,f, Tables S8, S9).

### 3. Single‐nucleus analysis captures more accurate cell type composition and transcriptome profiles

**Fig. 3:**
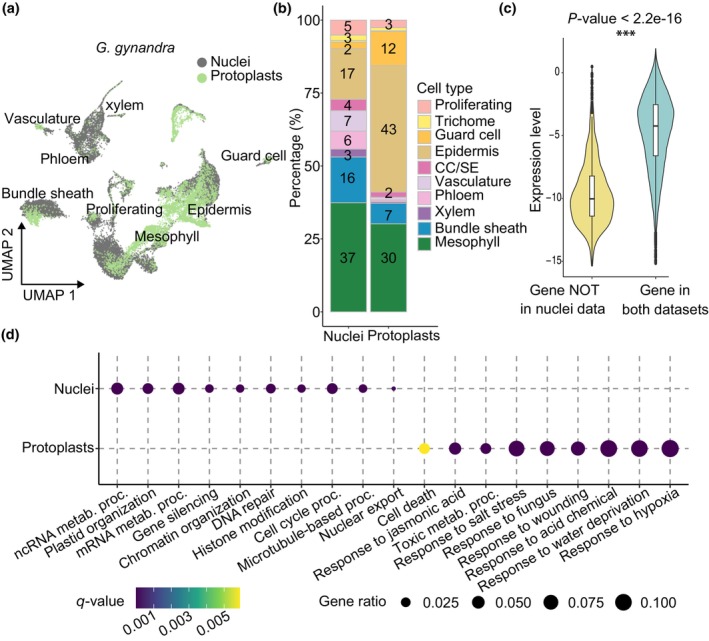
Comparison between single‐nucleus and single‐protoplast transcriptomes from *Gynandropsis gynandra* leaves. (a) Uniform Manifold Approximation and Projection (UMAP) visualisation of combined protoplast and nucleus transcriptomes. Major clusters annotated by cell type. Nuclei shown as grey and protoplasts as green. (b) Bar chart showing representation of each cell type estimated from nucleus or protoplast data based on expression of marker genes. CC/SE, companion cell/sieve element related cell. (c) Violin plot illustrating expression of genes detected only in protoplasts (yellow) or both nuclei and protoplasts (teal). The shape of each violin represents the kernel density estimate, and the horizontal line indicates the median value. The box spans the interquartile range (IQR; 25^th^–75^th^ percentile). Whiskers extend to the most extreme data points within 1.5 IQR from the quartiles, and the points beyond the whiskers are plotted as outliers. Asterisks indicate a *t*‐test *P*‐value smaller than 0.01 between the two groups, with the exact values also shown in the plot (d) Dot plots showing enriched Gene Ontology (GO) terms for genes that were differentially expressed between nuclei and protoplasts. Metab., metabolic; proc., process.

Once data had been integrated, patterns of gene expression derived from nuclei and protoplasts as depicted by UMAPs showed reasonable congruence (Figs 3a, S7a) and marker genes for each cell type exhibited similar patterns of expression (Figs S7b, S8a). Thus, single‐nucleus and single‐protoplast sequencing captured comparable transcriptome profiles. However, while both approaches allowed us to detect all major cell types of the leaf, including mesophyll, bundle sheath, epidermis, guard cells, proliferating cells and vascular cells, the abundance of these cell types differed between the two approaches. For example, although a similar percentage of cells detected were annotated as mesophyll, nuclei had a greater representation of internal cells, such as vascular cells and the bundle sheath, and much lower proportions of guard and epidermal pavement cells compared with protoplasts (Fig. 3b). Notably, in *G. gynandra* the most abundant cell type detected after protoplasting were epidermis cells (43% of all cells). Similar trends were observed for *F. bidentis* (Fig. S7c). Although the relative abundance of each cell type varied between methods and species, these patterns were largely consistent across replicates, suggesting that the observed differences reflect systematic biases rather than sampling noise (Fig. S8b,c). Although some differences in cell type composition between datasets may be explained by species differences or different developmental stages, in general the single‐nucleus datasets appeared to more accurately reflect the cell type composition of a leaf and captured internal cells that are less likely to be digested by cell wall degrading enzymes.

Fewer genes were detected in nuclei compared with protoplasts, but this effect could be offset by sequencing more nuclei such that the total number of genes detected was similar between these datasets (Figs S7d, S8d). Genes not detected by single‐nucleus sequencing were primarily poorly expressed genes (Figs 3c, S7e,f, S8e). Analysis of differences in transcript abundance between nuclei and protoplasts in *G. gynandra* highlighted that the nuclei data mostly captured genes associated with RNA, DNA organisation and gene expression, while protoplast data captured many genes related to stress responses (Figs 3d, S9a,c; Tables S10, S11). This was also the case in *F. bidentis* (Figs S7h, S9b,d; Tables S12, S13). Overall, we conclude that both single‐nucleus and single‐protoplast data allow identification of major cell types from C_4_ leaves, but analysis of nuclei appeared to provide two advantages. First, the representation of different cell types was more comparable to the proportion of each cell type found in the leaf, and second, there was less evidence for upregulation of stress response pathways. We therefore chose to analyse the single‐nucleus data from *G. gynandra* and *F. bidentis* to better understand similarities and differences in gene expression between these C_4_ plants.

### 4. Conserved patterning of structural genes as well as transcription factors in two different C_4_ subtypes

**Fig. 4:**
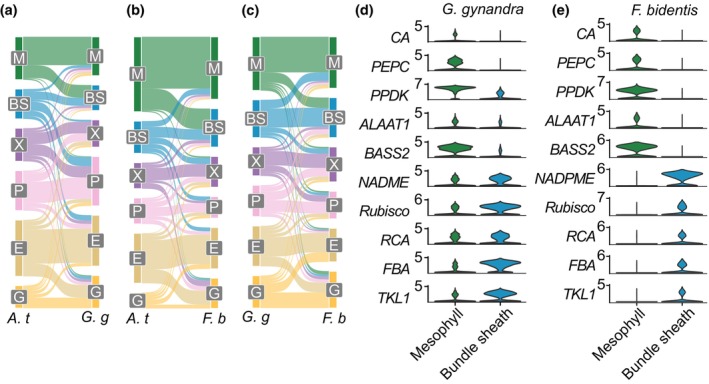
Compartmentalisation of genes between bundle sheath and mesophyll. (a–c) Sankey plots summarising changes in compartmentalisation of gene expression between C_3_ *Arabidopsis thaliana* and C_4_ *Gynandropsis gynandra* (a), C_3_ *A. thaliana* and C_4_ *Flaveria bidentis* (b), and C_4_ *G. gynandra* and C_4_ *F. bidentis* (c). *A. t*., *A. thaliana*; *G. g*., *G. gynandra*; *F. b*., *F. bidenti*s. BS, bundle sheath; E, epidermis; G, guard cell; M, mesophyll; P, phloem; X, xylem. Connections indicate shared marker genes so that link widths are proportional to the number of overlapping genes. (d, e) Violin plots showing transcript abundance of genes encoding key enzymes and transporters involved in the C_4_ cycle and Calvin–Benson–Bassham (CBB) cycle genes in mesophyll (green) and bundle sheath (blue) cells of *G. gynandra* (d) and *F. bidentis* (e). Each violin represents the distribution of expression values for a given gene across all cells of a specific type. The width of the violin reflects the density of cells expressing at that level. Gene names are shown on the *y*‐axis, and cell types are indicated on the *x*‐axis and by colour.

To provide context for the analysis of the single‐nucleus data from *G. gynandra* and *F. bidentis*, we first compared each with publicly available data from the C_3_ model *Arabidopsis thaliana* (Kim *et al*., 2021). The presence or absence of top marker genes of each cell type was compared between the species. In both cases, the strongest connections were observed between the same cell types, indicating a significant amount of conservation between the patterns of gene expression in cell type (Fig. 4a,b). However, bundle sheath cells from *G. gynandra* and *F. bidentis* shared greater similarity with mesophyll cells from *A. thaliana* than with the bundle sheath and were more similar to each other than to these cells from C_3_ *A. thaliana* (Fig. 4a–c). This similarity was associated with genes involved in the light‐dependent reactions of photosynthesis (e.g. LHCA1: OG0009556, LHCA3: OG0009361 and LHCA4: OG0007442), the Calvin–Benson–Bassham cycle (e.g. FBA: OG0001032) and photorespiration (including subunits of the glycine decarboxylase complex, GLDT: OG0008599 and GLDH: OG0008404, as well as SHM1: OG0002906; see Tables S4, S5 for cell type markers, and Table S14 for their orthology). Thus, despite diverging from a common ancestor *c*. 100 Ma and employing different C_4_ acid decarboxylases to release CO_2_ around RuBisCO, *G. gynandra* and *F. bidentis* exhibited similar patterns of gene expression in bundle sheath cells.

As would be expected, core C_4_ genes showed similar patterns of expression in both species (Fig. 4d,e), and photorespiration genes were rewired to be strongly expressed in the bundle sheath (Fig. S10). Additionally, several transporter, nitrogen assimilation and amino acid metabolism genes also displayed cell type preferential expression (Figs S11, S12). Whereas the expression of genes encoding enzymes of photorespiration needs to be downregulated in C_4_ plants compared with the ancestral C_3_ state, those of the C_4_ cycle are upregulated in either mesophyll or bundle sheath cells. Most C_4_ cycle genes are encoded by multigene families, and so we investigated whether particular gene copies are recruited into C_4_ photosynthesis in both *G. gynandra* and *F. bidentis*. For most proteins, in both species gene copies from one orthogroup were upregulated in either mesophyll or bundle sheath. This included genes encoding a chloroplastic PYROPHOSPHATASE from OG0007017 needed for PPDK to function in mesophyll cells (Figs S13, 14; Table S15). However, we also found evidence that convergent evolution underpins the recruitment of C_4_ cycle enzymes in these species. For example, for both *PEPC* and *ADENYLATE KINASE (AMK)* different gene copies became strongly expressed in mesophyll cells of *G. gynandra* and *F. bidentis* (Figs S15, S16). Moreover, in the case of *CARBONIC ANHYDRASE (CA)* where previous analysis indicated that the loss of a chloroplast transit peptide allowed increased activity in the cytosol of mesophyll cells of *Flaveria* (Tanz *et al*., 2009) and also *Neurachne* (Clayton *et al*., 2017), in *G. gynandra* we found no evidence for this (Fig. S17; Table S16). Instead, in *G. gynandra*, transcripts encoding a cytosolic CA isoform were upregulated in mesophyll cells.

The data also allowed bundle sheath and other vascular cells to be separated from each other, and so provide insight into patterns of gene expression specifically associated with the bundle sheath. For example, in both species, genes preferentially expressed in the mesophyll were also generally highly expressed in epidermis and guard cells, whereas genes preferentially expressed in the bundle sheath were often highly expressed in the xylem or phloem (Fig. S18). Previous work established that the three C_4_ acid decarboxylases (NADP‐ME, NAD‐ME and PEPCK) that provide CO_2_ to RuBisCO in C_4_ plants are preferentially expressed in vascular cells of C_3_ species (Brown *et al*., 2010; Hibberd & Quick, 2002). Here, we investigated whether expression of these genes was detected in phloem and/or xylem cells of *G. gynandra* and *F. bidentis*. In *G. gynandra*, both *NAD‐ME* genes showed strong expression in the bundle sheath, and *NAD‐ME1* was also detected in phloem cells. In *F. bidentis*, one *NADP‐ME* gene was strongly expressed in the bundle sheath, but was barely detected in the vasculature (Figs S19, S20). *PEPCK* did not show strong expression in *G. gynandra* bundle sheath, but was detected in the phloem (Fig. S21).

**Fig. 5:**
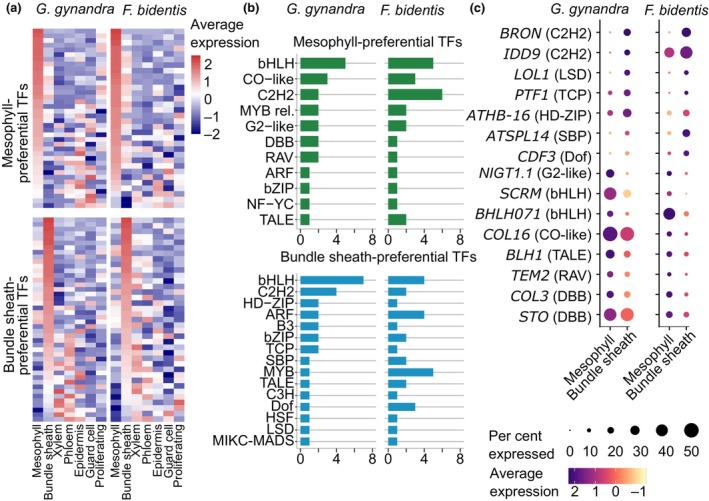
Conserved patterning of transcription factors between bundle sheath and mesophyll cells. (a) Heatmap of transcription factors that were differentially expressed between mesophyll and bundle sheath cells of *Gynandropsis gynandra* (left) and *Flaveria bidenti*s (right). (b) Number of transcription factors by family showing preferential expression in mesophyll or bundle sheath cells in *G. gynandra* (left) and *F. bidentis* (right), MYB rel., MYB‐related. (c) Transcript abundance of orthologous transcription factors that were preferentially expressed in either mesophyll or bundle sheath cells of both *G. gynandra* (left) and *F. bidentis* (right).

To better understand how signatures of bundle sheath and mesophyll cells may be specified, we next explored transcription factors that were preferentially expressed in each cell type. Thirty‐eight mesophyll and forty‐four bundle sheath‐preferential transcription factors were identified in *G. gynandra*, while in *F. bidentis*, 32 mesophyll and 37 bundle sheath‐preferential transcription factors were detected (Fig. 5a). As with marker genes, transcription factors preferentially expressed in the mesophyll were also highly expressed in the epidermis and guard cells, while those enriched in the bundle sheath exhibited stronger expression in vascular tissues. Families such as the bHLH, C2H2 and homeobox transcription factors had genes that were specific to mesophyll and bundle sheath cells in both *G. gynandra* and *F. bidentis* (Fig. 5b; Table S17). However, it was notable that in both species, members of the DOF and IDD families were specific to bundle sheath cells (Figs 5b, S22). These were also identified as bundle sheath specific from the protoplast data (Fig. S23). Moreover, we identified 15 transcription factors belonging to the same orthogroups that were specific to either the mesophyll or bundle sheath cells from both species (Fig. 5c). This included seven that were bundle sheath preferential and eight that were mesophyll preferential. The top two most strongly expressed of these in the bundle sheath (BRON and IDD9) belonged to the C_2_H_2_ family. To further investigate gene expression patterns associated with these transcription factors, we constructed co‐expression networks for both *G. gynandra* and *F. bidentis* (Fig. S24; Tables S18, S19). In both species, *BRON* ranked among the top five hub transcription factors within a module associated with bundle sheath identity. Interestingly, the bundle sheath‐preferential *IDD9* in *G. gynandra* did not fit into any module, whereas in *F. bidentis*, it grouped with genes that also related to proliferating cells. Other shared mesophyll‐preferential regulators, such as *NIGT1.1* and *SCRM*, were also found to be highly connected within their respective modules. These results provide new candidates to test for roles in transcriptional patterning in leaves.

In summary, we report optimisation of both single‐nucleus and single‐protoplast RNA‐seq and, as a consequence, construct high‐resolution transcriptome atlases for two independently evolved C_4_ species. Our analysis indicates that single‐nucleus sequencing better preserves native cell type composition, and transcriptome profiles showed lower signatures of stress. Using the nuclei data, we confirmed core C_4_ gene compartmentalisation and identified transcriptional signatures of mesophyll and bundle sheath cells. Furthermore, we found that in both species, mesophyll‐enriched genes were often co‐expressed in epidermal and guard cells, suggesting some degree of shared regulation between these cell types. By contrast, bundle sheath‐enriched genes overlapped more with xylem and phloem, pointing to a closer transcriptional relationship with these vascular tissues. Together, our findings provide new insights into the spatial partitioning of gene expression needed for C_4_ photosynthesis and serve as a valuable resource for future studies on the evolution and engineering of C_4_ traits.

## IV. Discussion

After direct comparison between single‐protoplast and single‐nucleus sequencing, we propose that the latter provides a more accurate representation of cell type composition and more effectively characterises native gene expression across different cell types from leaves. Both approaches validated known patterns of gene expression associated with C_4_ photosynthesis. However, by capturing transcriptomes at single‐cell or single‐nucleus resolution, we were also able to separate signatures from bundle sheath cells and vascular tissues, and by including two independently evolved C_4_ species, identified transcription factors not previously associated with bundle sheath cells. These cell‐specific atlases provide valuable resources to study gene compartmentalisation in the context of leaves in general and C_3_ and C_4_ photosynthesis in particular.

To generate high‐quality transcriptome atlases at cellular resolution for leaves of two C_4_ dicotyledons, we employed various methods to optimise protoplast and nuclei isolation protocols tailored for the most widely used single‐cell platforms. As previously reported (Sawers *et al*., 2007; Chupeau *et al*., 2013), protoplast isolation led to transcriptome signatures associated with stress. Because of this sensitivity of leaf transcriptomes to abiotic stress during protoplast isolation, despite nuclei‐based analysis detecting fewer transcripts, we conclude that their analysis is informative as stress‐related signals are lower, and that the proportion of each cell type detected is closer to the tissue composition of the leaf. Because of these advantages, we primarily focused on the analysis of the nuclei data to investigate gene expression in the two C_4_ dicotyledons: *G. gynandra* and *F. bidentis*. In contrast to C_4_ species in the monocotyledons (Bezrutczyk *et al*., 2021; Swift *et al*., 2024), although both *G. gynandra* and *F. bidentis* are used as models to understand C_4_ photosynthesis (Brown *et al*., 2011; Gowik *et al*., 2011; Schulze *et al*., 2013; Hoang *et al*., 2023; Singh *et al*., 2023; Schreier *et al*., 2024) single‐cell data have previously not been available. Thus, our analysis provides transcriptome data not only for bundle sheath and mesophyll cells but also for other critical cell types, such as vascular and guard cells, which play essential roles in C_3_ and C_4_ leaves. Although these two C_4_ species diverged from a common ancestor over 100 Ma (Moore *et al*., 2010; Zuntini *et al*., 2024), we find that the transcriptome profile of bundle sheath cells in *G. gynandra* shares greater similarity with *F. bidentis* than with those available from the more closely related C_3_ model, *A. thaliana*, which diverged from a common ancestor with *G. gynandra* 41 Ma (Schranz & Mitchell‐Olds, 2006). Previous single‐nucleus analysis of the monocotyledons rice and sorghum revealed that all cell types showed differences in gene expression between these C_3_ and C_4_ species, but the bundle sheath cells showed the most significant rewiring (Swift *et al*., 2024). Thus, combined with this previous work, the data reported here indicate that the evolution of the C_4_ pathway in the dicotyledons as well as the monocotyledons has resulted in major rewiring of the bundle sheath. Even when distinct biochemistries are being used to decarboxylate CO_2_ in the bundle sheath cells of these two species, their transcriptomes showed greater convergence to each other than to bundle sheath cells from C_3_ *A. thaliana*.

To better understand the basis for this convergence in bundle sheath gene expression in *G. gynandra* and *F. bidentis*, we investigated transcription factors that were preferentially expressed in these cells in both species. To our knowledge, there are only two examples where transcription factors that are both necessary and sufficient for bundle sheath expression have been defined. The first comprises a bipartite MYC‐MYB complex in *A. thaliana* (Dickinson *et al*., 2020) that has now been implicated in the evolution of C_2_ photosynthesis (Dickinson *et al*., 2025). The second includes a collective of transcription factors from WRKY, G2‐like, DOF, MYB‐related, IDD and bZIP families in rice (Hua *et al*., 2025; Swift *et al*., 2024). Interestingly, in the data we report here members of transcription factors from the MYB but not the MYC family were bundle sheath preferential in both *F. bidentis* and *G. gynandra*. This is the same patterning of expression reported for *A. thaliana*, where MYBs rather than MYCs drive cell specificity (Dickinson *et al*., 2020). Moreover, although first reported in monocotyledonous species (Hua *et al*., 2025; Swift *et al*., 2024) the single‐nucleus sequencing reported here now reveals that transcription factors from the DOF, IDD and bZIP families are preferentially expressed in bundle sheath cells of *F. bidentis* and *G. gynandra*. In rice, DOF and IDD motifs are also enriched in the bundle sheath (Swift *et al*., 2024); thus, it is possible that a team of transcription factors based on these core members mediates bundle sheath expression in dicotyledons. In addition to these previously reported regulators of bundle sheath expression, we also identified previously unknown transcription factor families and orthogroups that were preferentially expressed in this cell type in both *F. bidentis* and *G. gynandra*. This included C_2_H_2_, LSD, HD‐ZIP and SBP transcription factors. Currently available single‐nucleus data from C_3_ models in the dicotyledons, such as *A. thaliana* (Kim *et al*., 2021; Lopez‐Anido *et al*., 2021; Tenorio Berrio *et al*., 2022), have not been performed at sufficient depth to determine whether these genes are also preferentially expressed in bundle sheath cells in the ancestral C_3_ state. Furthermore, although transformation of *F. bidentis* and *G. gynandra* is possible, it is relatively laborious (Chitty *et al*., 1994; Newell *et al*., 2010), and so functional analysis confirming an unequivocal role of these transcription factors in cell specificity will need to be proved in the future. To address these questions, future studies that obtain single‐nucleus RNA‐seq data from close C_3_ relatives of these species will be helpful. Moreover, analysis of, for example, the *Flaveria* genus that contains intermediate photosynthetic types (that appear to span a trajectory from C_3_, to C_3_–C_4_, to C_2_ and C_4_ photosynthesis; Sage *et al*., 2012) is also likely to inform us about the rewiring of both mesophyll and bundle sheath cells during the evolution of C_4_ photosynthesis. Recent work using single‐nucleus RNA‐seq of a C_3_–C_4_ intermediate, *Moricandia arvensis*, highlighted functional specialisation of bundle sheath cells in the photorespiratory shuttle, which exemplifies the power of such approaches (Triesch *et al*., 2025).

In summary, our single‐cell resolution *trans*criptome atlases for *G. gynandra* and *F. bidentis* leaves provide the first comprehensive maps for gene expression at this level for C_4_ dicotyledons. They capture all major cell types in the leaf and also provide the quantification of gene expression in each of these cell types. To maximise the accessibility and utility of this atlas, we developed a web‐based portal that is user‐friendly and interactive for data visualisation and exploration. The portal includes side‐by‐side UMAP plots to allow comparison between gene expression and cell annotation, proportion plots to assess cell group composition, and dot plots and heatmaps to visualise transcripts derived from multiple genes across selected cell groups. By facilitating efficient exploration of these complex datasets, our web portal empowers the research community to fully utilise and build upon the atlas.

## Competing interests

None declared.

## Author contributions

JMH, LL and TS conceived and directed the research. TS, LL and JMH designed the experiments. TS and LL performed the single‐protoplast and single‐nucleus RNA‐seq experiments. TS analysed the single‐protoplast and single‐nucleus RNA‐seq data. VSB and BS performed the genome‐related experiments and analyses. TS, LL and JMH wrote the manuscript with input from all authors.

## Disclaimer

The New Phytologist Foundation remains neutral with regard to jurisdictional claims in maps and in any institutional affiliations.

## Supporting information


**Fig. S1** Workflow illustrating sampling and isolation of nuclei and protoplasts for sequencing.
**Fig. S2** Representative FACS plots of nuclei sorting.
**Fig. S3** Optimisation of protoplast isolation for single‐protoplast RNA‐seq.
**Fig. S4** Clustering and cell type annotation of single‐nucleus and single‐protoplast RNA sequencing from leaves of *G. gynandra* and *F. bidentis*.
**Fig. S5** Cluster annotation for the integrated transcriptome atlases of *G. gynandra* and *F. bidentis* leaves.
**Fig. S6** Number of cell type marker genes shared across different cell types.
**Fig. S7** Comparison of single‐nucleus and single‐protoplast transcriptome profiles in *F. bidentis* leaves.
**Fig. S8** Comparison of gene detection between single‐nucleus and single‐protoplast transcriptome profiles from *G. gynandra* leaves and cell type representation across replicates.
**Fig. S9** Comparison of stress and nuclei signature scores.
**Fig. S10** Expression of photorespiration genes.
**Fig. S11** Expression of transporter genes.
**Fig. S12** Expression of nitrogen assimilation and amino acid metabolism genes.
**Fig. S13** Phylogenetic reconstruction of the species used to identify orthogroups.
**Fig. S14** Phylogenetic tree of the PPA orthogroups.
**Fig. S15** Phylogenetic tree of the PEPC orthogroup.
**Fig. S16** Phylogenetic tree of the AMPK orthogroup.
**Fig. S17** Phylogenetic tree of the CA orthogroup.
**Fig. S18** Expression of differentially expressed genes between mesophyll and bundle sheath.
**Fig. S19** Phylogenetic tree of the NAD‐ME orthogroups.
**Fig. S20** Phylogenetic tree of the NADP‐ME orthogroup.
**Fig. S21** Phylogenetic tree of the PEPCK orthogroup.
**Fig. S22** Number of all mesophyll and bundle sheath‐preferential transcription factors characterised into different families in *G. gynandra* and *F. bidentis*.
**Fig. S23** Compartmentation of transcription factors between bundle sheath and mesophyll using only protoplast data.
**Fig. S24** UMAP projection of the gene co‐expression network and expression patterns of module eigengenes.

**Table S1** Assessment of contiguity and completeness of the reference‐quality assembly.
**Table S2** Amount of genome sequence data generated using each type of sequencing platform.
**Table S3** List of marker genes used for cell type annotation.
**Table S4** List of marker genes for each cell type in *G. gynandra*.
**Table S5** List of marker genes for each cell type in *F. bidentis*.
**Table S6** Best BLAST hit of *G. gynandra* genes in *A. thaliana*.
**Table S7** Best BLAST hit of *F. bidentis* genes in *A. thaliana*.
**Table S8** GO enrichment analysis of *A. thaliana* orthologs of *G. gynandra* cell type marker genes.
**Table S9** GO enrichment analysis of *A. thaliana* orthologs of *F. bidentis* cell type marker genes.
**Table S10** List of differentially expressed genes between protoplast (avg_log2FC > 0) and nuclei in *G. gynandra*.
**Table S11** GO enrichment analysis of *A. thaliana* orthologs of differentially expressed genes between protoplasts and nuclei in *G. gynandra*.
**Table S12** List of differentially expressed genes between protoplast (avg_log2FC > 0) and nuclei in *F. bidentis*.
**Table S13** GO enrichment analysis of *A. thaliana* orthologs of differentially expressed genes between protoplasts and nuclei in *F. bidentis*.
**Table S14** Orthogroup description for top marker genes shared between *G. gynandra* and *F. bidentis* (deduplicated).
**Table S15** TargetP prediction of the subcellular localisation of PPAs.
**Table S16** TargetP prediction of the subcellular localisation of CAs.
**Table S17** Differentially expressed transcription factors in mesophyll and bundle sheath cells.
**Table S18** Co‐expression modules in *G. gynandra*.
**Table S19** Co‐expression modules in *F. bidentis*.
Please note: Wiley is not responsible for the content or functionality of any Supporting Information supplied by the authors. Any queries (other than missing material) should be directed to the *New Phytologist* Central Office.

## Data Availability

All data generated in this study are publicly available. The genome sequence data reported in this paper are available at NCBI under BioProject ID PRJNA1259992, and the genome assembly and annotation can be accessed at: https://figshare.com/s/7fb9444d7cefef0c341f. The single‐protoplast and single‐nucleus sequencing data are available at ArrayExpress under accession no.: E‐MTAB‐15121. Our atlases can be explored via online web portals: https://hibberdlab.shinyapps.io/gg_integrated/ for *G. gynandra* and https://hibberdlab.shinyapps.io/fb_integrated/ for *F. bidentis*. The code generated during this study is available at GitHub: https://github.com/TianshuSunCam/C4_dicots_single_nucleus_protoplast.
