# Supplementary material for: Conserved spatial patterning of gene expression in independent lineages of C4 plants
Source: New Phytol. 2025 Aug 22;249(1):24–38. doi: 10.1111/nph.70475 (PMC12676079; doi:10.1111/nph.70475)

## *New Phytologist* Supporting Information

Article title: Conserved spatial patterning of gene expression in independent lineages of C_4_ plants

Authors: Tianshu Sun, Venkata Suresh Bonthala, Benjamin Stich, Leonie Luginbuehl, Julian M. Hibberd

Article acceptance date: 16 July 2025

The following Supporting Information is available for this article:

**Fig. S1** Workflow illustrating sampling and isolation of nuclei and protoplasts for sequencing.

**Fig. S2** Representative FACS plots of nuclei sorting.

**Fig. S3** Optimisation of protoplast isolation for single-protoplast RNA-seq.

**Fig. S4** Clustering and cell type annotation of single-nucleus and single-protoplast RNA sequencing from leaves of *G. gynandra* and *F. bidentis*.

**Fig. S5** Cluster annotation for the integrated transcriptome atlases of *G. gynandra* and *F. bidentis* leaves.

**Fig. S6** Number of cell type marker genes shared across different cell types.

**Fig. S7** Comparison of single-nucleus and single-protoplast transcriptome profiles in *F. bidentis* leaves.

**Fig. S8** Comparison of gene detection between single-nucleus and single-protoplast transcriptome profiles from *G. gynandra* leaves and cell type representation across replicates.

**Fig. S9** Comparison of stress and nuclei signature scores.

**Fig. S10** Expression of photorespiration genes.

**Fig. S11** Expression of transporter genes.

**Fig. S12** Expression of nitrogen assimilation and amino acid metabolism genes.

**Fig. S13** Phylogenetic relationship of the species used to identify orthogroups.

**Fig. S14** Phylogenetic tree of the *PPA* orthogroups.

**Fig. S15** Phylogenetic tree of the *PEPC* orthogroup.

**Fig. S16** Phylogenetic tree of the *AMK* orthogroup.

**Fig. S17** Phylogenetic tree of the *CA* orthogroup.

**Fig. S18** Expression of differentially expressed genes between mesophyll and bundle sheath.

**Fig. S19** Phylogenetic tree of the *NAD-ME* orthogroups.

**Fig. S20** Phylogenetic tree of the *NADP-ME* orthogroup.

**Fig. S21** Phylogenetic tree of the *PEPCK* orthogroup.

**Fig. S22** Number of all mesophyll and bundle sheath preferential transcription factors characterised into different families in *G. gynandra* and *F. bidentis*.

**Fig. S23** Compartmentation of transcription factors between bundle sheath and mesophyll cells using only protoplast data.

**Fig. S24** UMAP projection of the gene co-expression network and expression patterns of module eigengenes.

**Table S1** Assessment of contiguity and completeness of the reference-quality assembly.

**Table S2** Amount of genome sequence data generated using each type of sequencing platform.

**Table S3** List of marker genes used for cell type annotation.

**Table S4** List of marker genes for each cell type in *G. gynandra*.

**Table S5** List of marker genes for each cell type in *F. bidentis*.

**Table S6** Best BLAST hit of *G. gynandra* genes in *A. thaliana*.

**Table S7** Best BLAST hit of F*. bidentis* genes in *A. thaliana*.

**Table S8** GO enrichment analysis of *A. thaliana* orthologs of *G. gynandra* cell type marker genes.

**Table S9** GO enrichment analysis of *A. thaliana* orthologs of *F. bidentis* cell type marker genes.

**Table S10** List of differentially expressed genes between protoplasts (avg_log2FC>0) and nuclei in *G. gynandra*.

**Table S11** GO enrichment analysis of *A. thaliana* orthologs of differentially expressed genes between protoplasts and nuclei in *G. gynandra*.

**Table S12** List of differentially expressed genes between protoplasts (avg_log2FC>0) and nuclei in *F.* *bidentis*.

**Table S13** GO enrichment analysis of *A. thaliana* orthologs of differentially expressed genes between protoplasts and nuclei in *F. bidentis*.

**Table S14** Orthogroup description for top marker genes shared between *G. gynandra* and *F. bidentis* (deduplicated).

**Table S15** TargetP prediction of the subcellular localization of PPAs.

**Table S16** TargetP prediction of the subcellular localization of CAs.

**Table S17** Differentially expressed transcription factors in mesophyll and bundle sheath cells.

**Table S18** Co-expression modules in *G. gynandra*.

**Table S19** Co-expression modules in *F. bidentis*.

**Fig. S1** Workflow illustrating sampling and isolation of nuclei and protoplasts for sequencing. Leaves from *G. gynandra* and *F. bidentis* were collected to allow nuclei isolation through Fluorescence-activated cell sorting (FACS, top) or protoplast isolation through enzymatic digestion (bottom). Isolated nuclei and protoplasts were then subjected to single-cell RNA sequencing for downstream analysis.


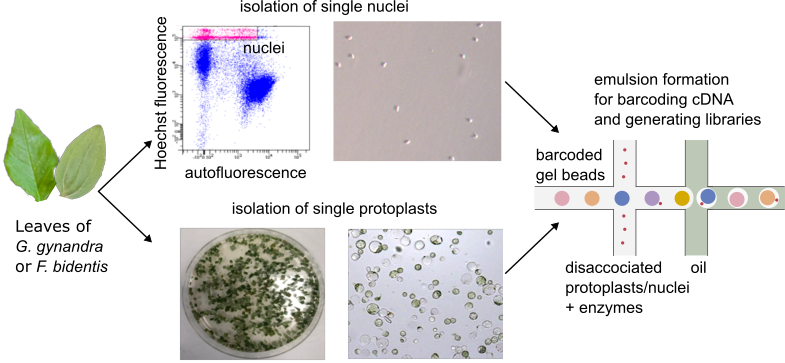


**Fig. S2** Representative FACS plots of nuclei sorting. The gate for unstained *G. gynandra* nuclei (a) and *F. bidentis* nuclei (d) preparations showing Hoechst fluorescence signals on the y-axis plotted against autofluorescence signals on the x-axis. The gate for Hoechst + *G. gynandra* nuclei is shown in red (b) and for Hoechst + *F. bidentis* nuclei in magenta (e). (c) and (f) show images of sorted nuclei under a light microscope.

**
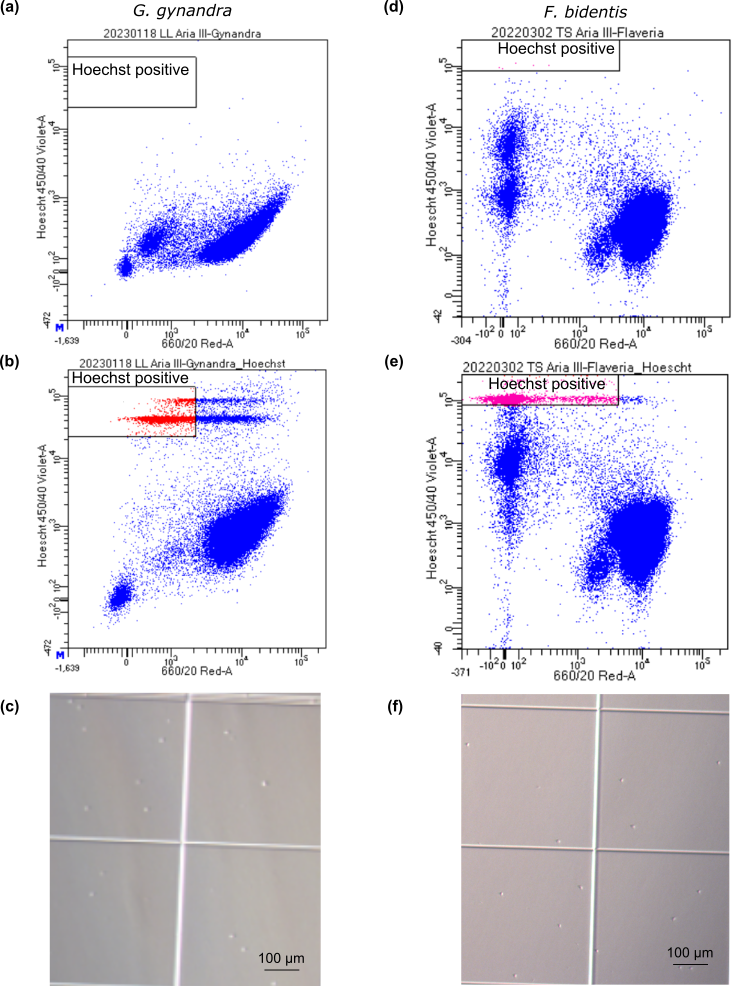
**

**Fig. S3** Optimisation of protoplast isolation for single-protoplast RNA-seq. Light microscopy images show isolated *F. bidentis* protoplasts derived from young leaves of a 5-week-old plant (a) and a 2-week-old plant (b). Isolated protoplasts resuspended in mannitol (c) or ½ W5 buffer (d). Successful cDNA synthesis and amplification were achieved in mannitol-resuspended protoplast samples (e), whereas no cDNA was amplified in W5-resuspended samples (f).


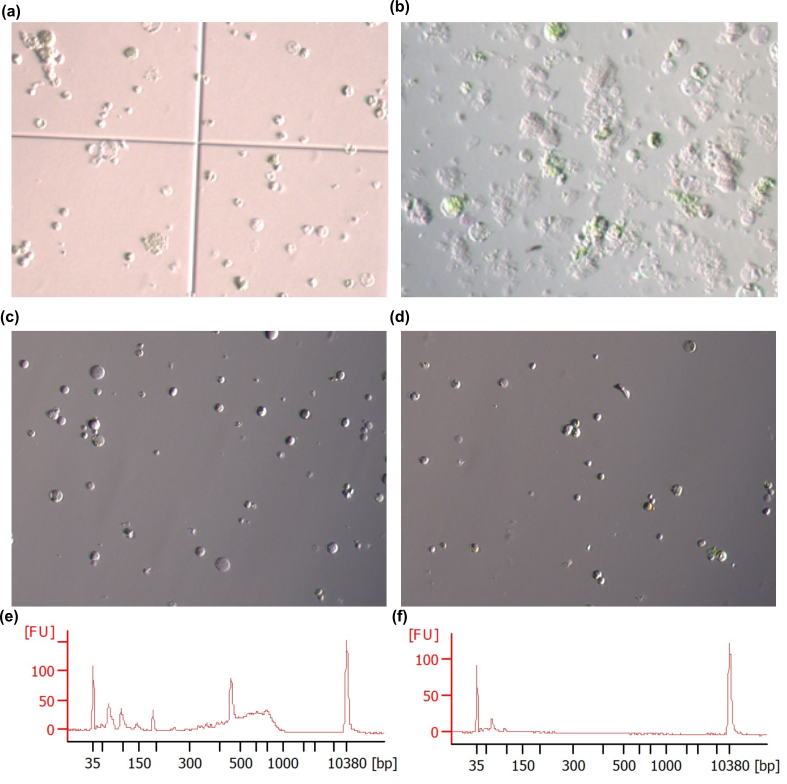


**Fig. S4** Clustering and cell type annotation of single-nucleus and single-protoplast RNA sequencing from leaves of *G. gynandra* and *F. bidentis*. (a-d) UMAP visualisation of the clustering of nuclei or protoplasts from *G. gynandra* (a) and (b) or *F. bidentis* (c) and (d), coloured by unsupervised clusters. (e-h) UMAP visualisation of the clustering of nuclei or protoplasts from *G. gynandra* (e) and (f) or *F. bidentis* (g) and (h), coloured by replicate. (i-l) Dot plots showing expression of marker genes defining major annotated cell types.


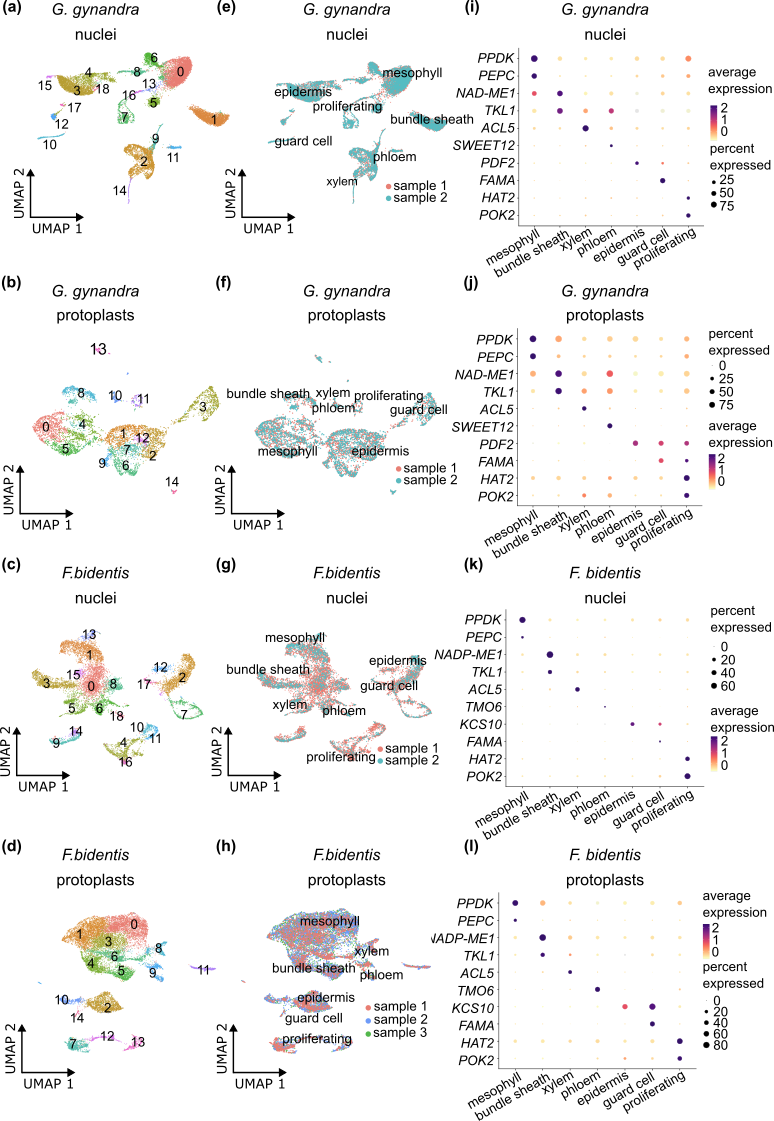


**Fig. S5** Cluster annotation for the integrated transcriptome atlases of *G. gynandra* and *F. bidentis* leaves. UMAP visualisation of combined nuclei and protoplast transcriptome profiles of *G. gynandra* (a) and *F. bidentis* (b), coloured by unsupervised clusters. Dot plots showing the expression of cell type-defining marker genes for each cluster in *G. gynandra* (c) and *F. bidentis* (d).


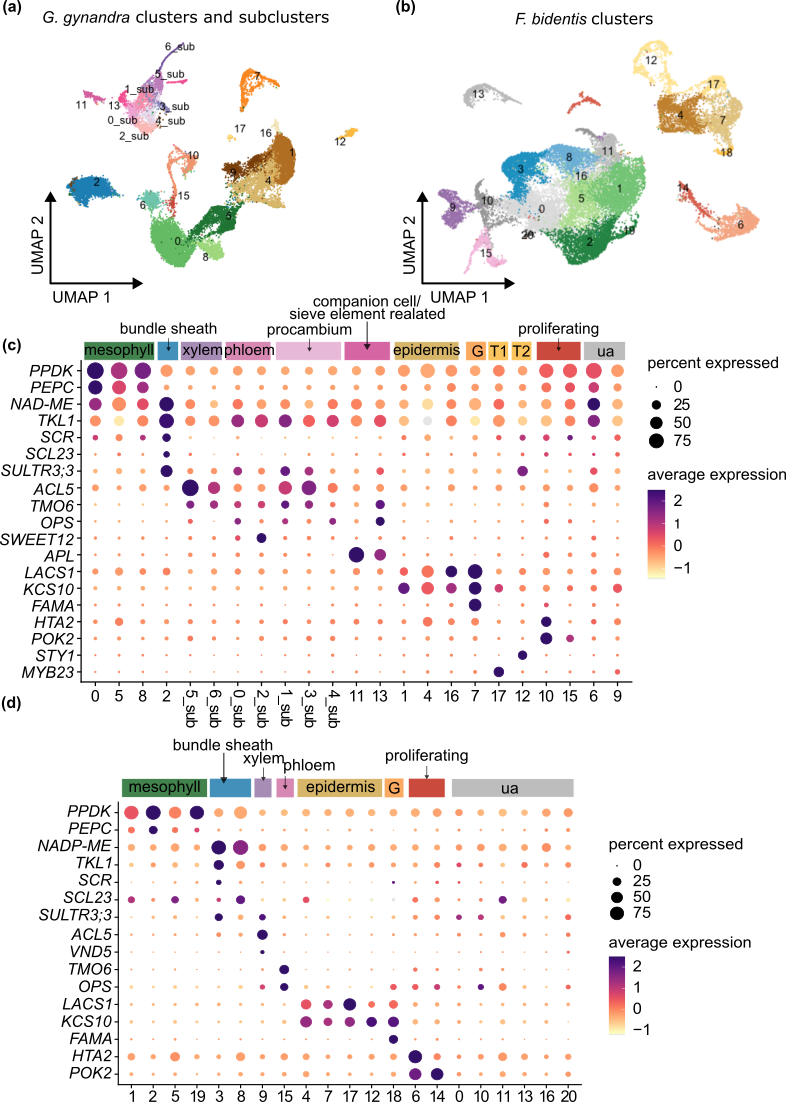


**Fig. S6** Number of cell type marker genes shared across different cell types. Upset plots illustrating the unique and shared top 200 marker genes across different cell types in *G. gynandra* (a) and *F. bidentis* (b).


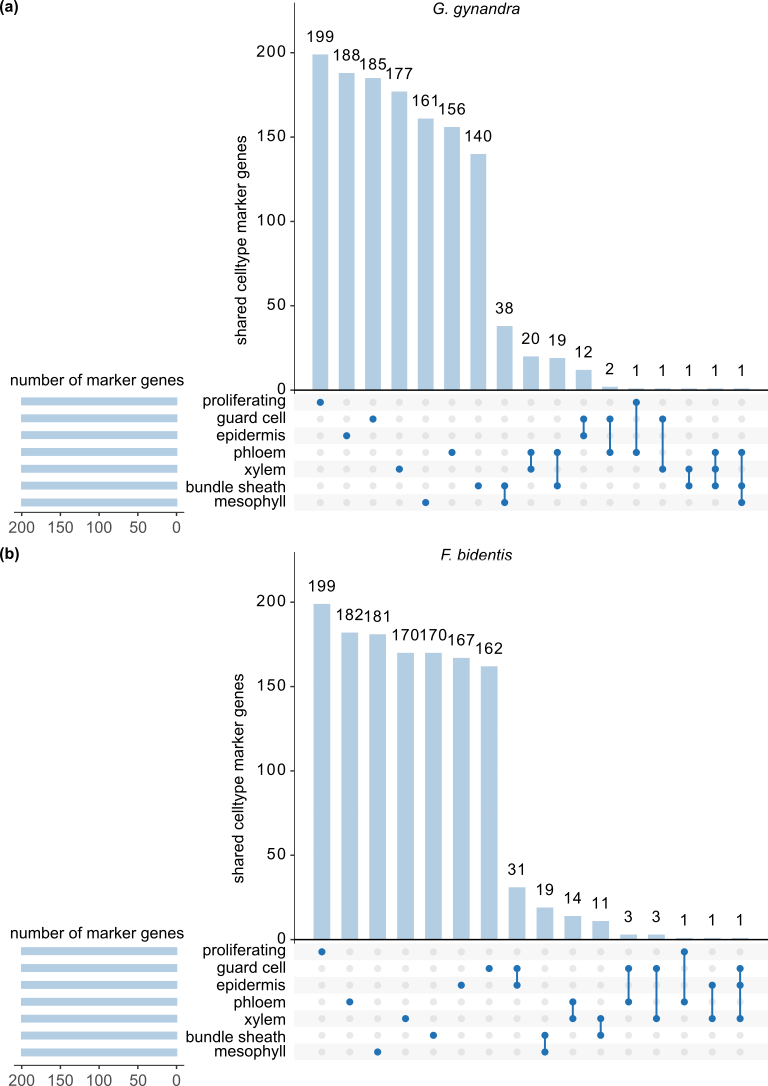


**Fig. S7** Comparison of single-nucleus and single-protoplast transcriptome profiles in *F. bidentis* leaves. (a) UMAP visualisation of combined protoplast and nuclei transcriptome profiles. Major clusters annotated by cell type. Nuclei shown as grey and protoplasts as green. (b) Dot plots showing the expression of marker genes for major cell types from either nuclei or protoplasts. (c) Bar chart showing representation of each cell type estimated from nucleus or protoplast data based on expression of marker genes. (d) Bar plot comparing the average number of genes per nucleus or protoplast, as well as the total number of genes detected in each profile.  (e) Density plot showing the distribution of gene ranks, where yellow represents genes detected only in the protoplast profile, and teal represents genes detected in both nuclei and protoplast profiles. (f) Violin plot illustrating expression of genes detected in nuclei only (yellow) or nuclei and protoplasts (teal). (g) Dot plots showing enriched Gene Ontology (GO) terms for genes that were differentially expressed between nuclei and protoplasts.


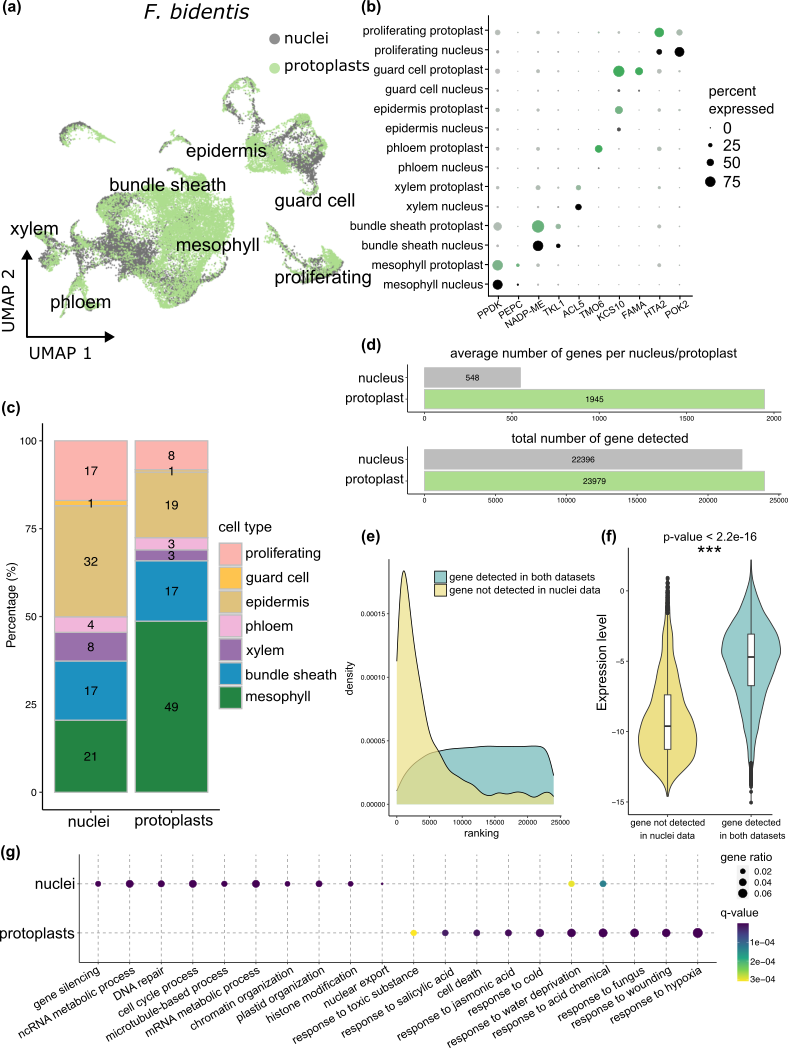


**Fig. S8** Comparison of gene detection between single-nucleus and single-protoplast transcriptome profiles from *G. gynandra* leaves and cell type representation across replicates. (a) Dot plots showing the expression of marker genes for major cell types from either nuclei or protoplasts. (b, c) Bar chart showing representation of each cell type estimated from nucleus or protoplast data based on expression of marker genes without (b) or with (c) the inclusion of unannotated cluster (ua) across replicates. (d) Bar plot comparing the average number of genes per nucleus or protoplast, as well as the total number of genes detected in each profile.  (e) Density plot showing the distribution of gene ranks, where yellow represents genes detected only in the protoplast profile, and teal represents genes detected in both nuclei and protoplast profiles.


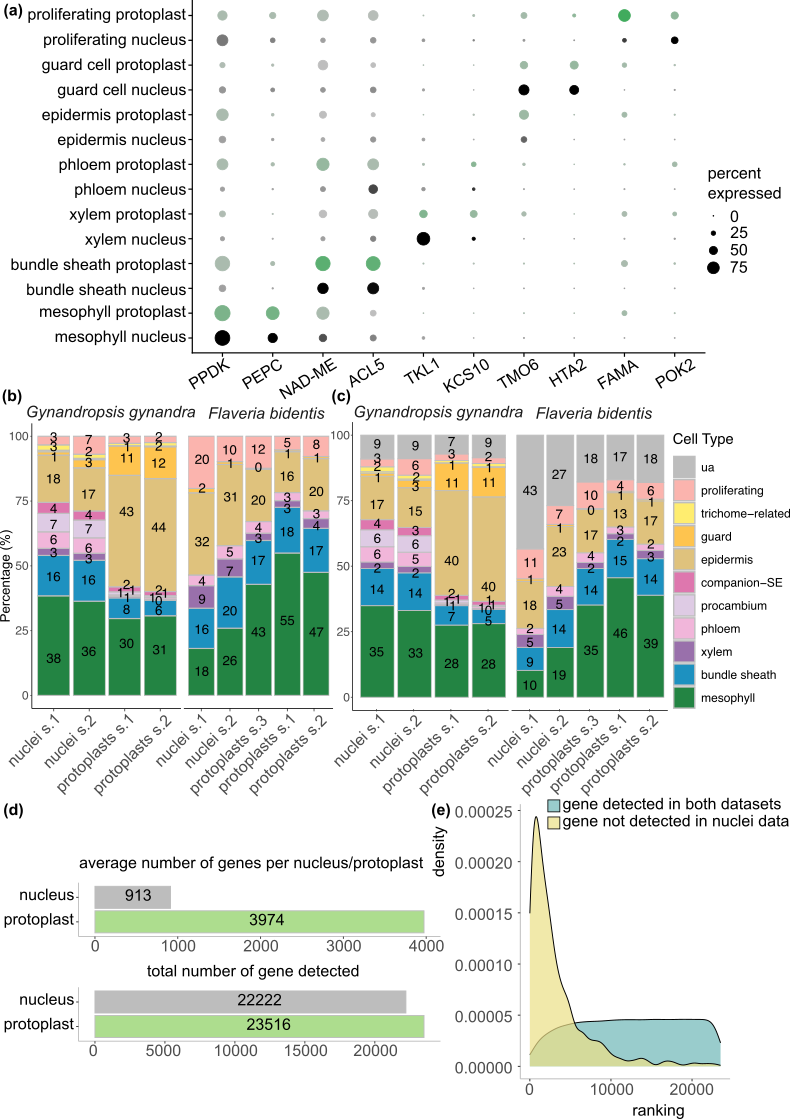


**Fig. S9** Comparison of stress and nuclei signature scores. Expression of the stress score in nucleus and protoplast data of *G. gynandra* (a) and *F. bidentis* (b). Expression of the nucleus score in nucleus and protoplast data of *G. gynandra* (c) and *F. bidentis* (d). In each comparison, the first panel (left) shows the expression of the signature score in the single-nucleus transcriptome profile, the middle panel shows its expression in the single-protoplast transcriptomic profile, and the last panel (right) compares the signature score between the two datasets using a violin plot. Statistical significance was assessed using the Wilcoxon rank-sum test, **** indicates p ≤ 0.0001.


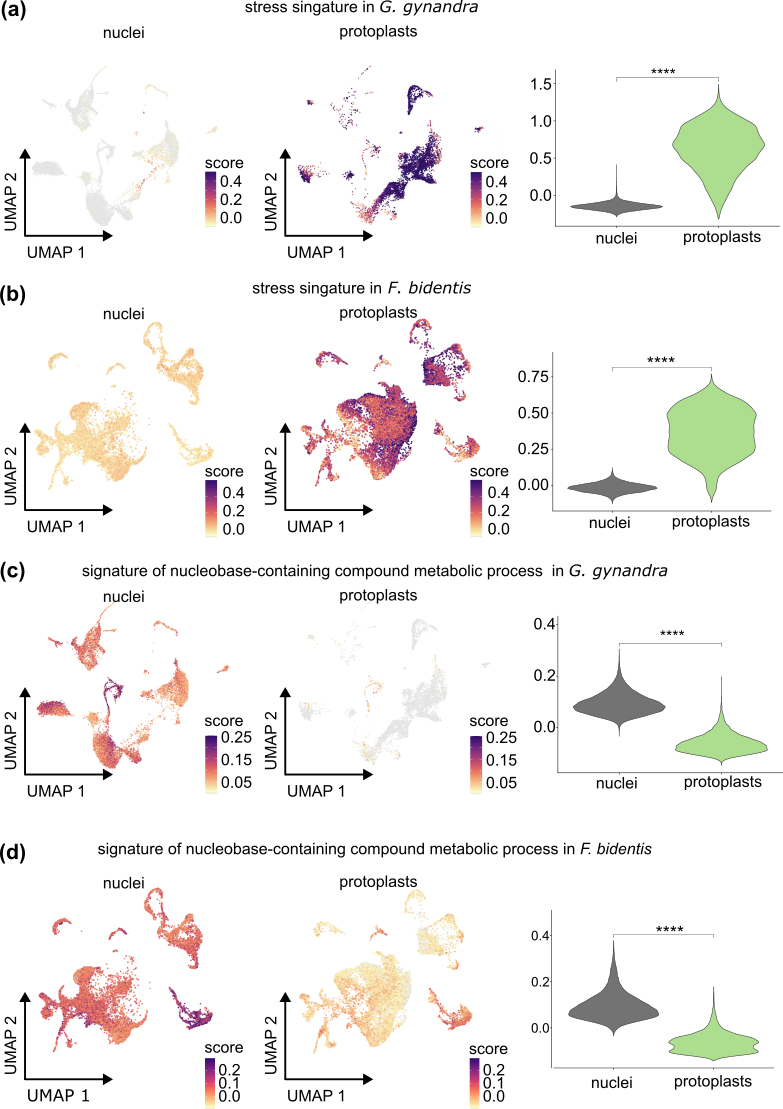


**Fig. S10** Expression of photorespiration genes. Dot plots showing transcript abundance of photorespiration genes across different cell types in *G. gynandra* (a) and *F. bidentis* (b). Gene copies with mesophyll-preferential expression are highlighted in green, and those with bundle sheath–preferential expression are highlighted in blue (p < 0.05). Genes with an adjusted p-value < 0.01 (Bonferroni correction) are shown in bold.


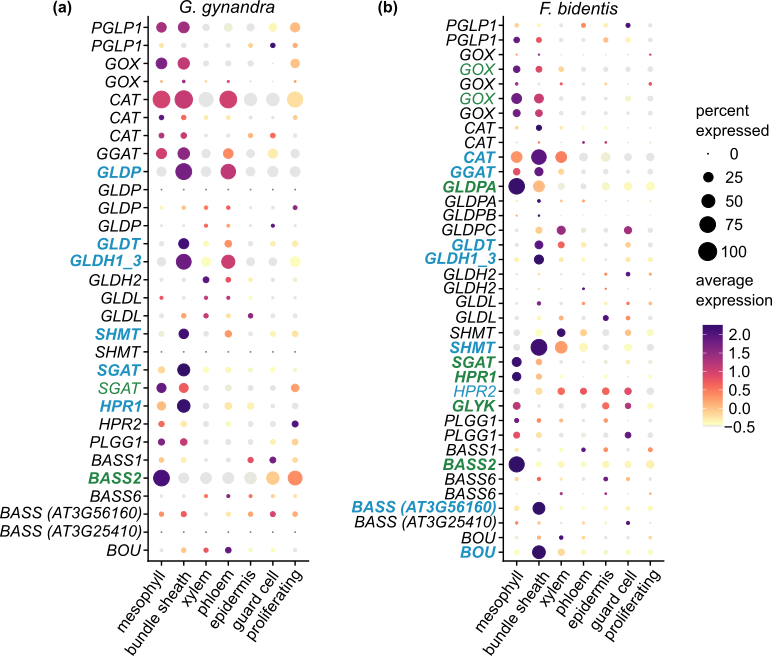


**Fig. S11** Expression of transporter genes. Dot plots showing transcript abundance of transporter genes across different cell types in *G. gynandra* (a) and *F. bidentis* (b). Gene copies with mesophyll-preferential expression are highlighted in green, and those with bundle sheath–preferential expression are highlighted in blue (p < 0.05). Genes with an adjusted p-value < 0.01 (Bonferroni correction) are shown in bold.


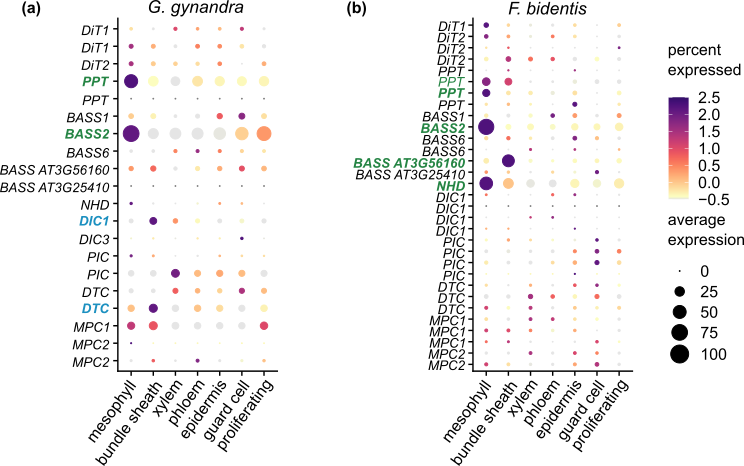


**Fig. S12** Expression of nitrogen assimilation and amino acid metabolism genes. Dot plots showing the expression of nitrogen assimilation and amino acid metabolism genes that are differentially expressed between mesophyll and bundle sheath cells in *G. gynandra* (a,c) and *F. bidentis* (b, d). Genes plotted above the dashed line are mesophyll-preferential, while those below the line are bundle sheath–preferential.


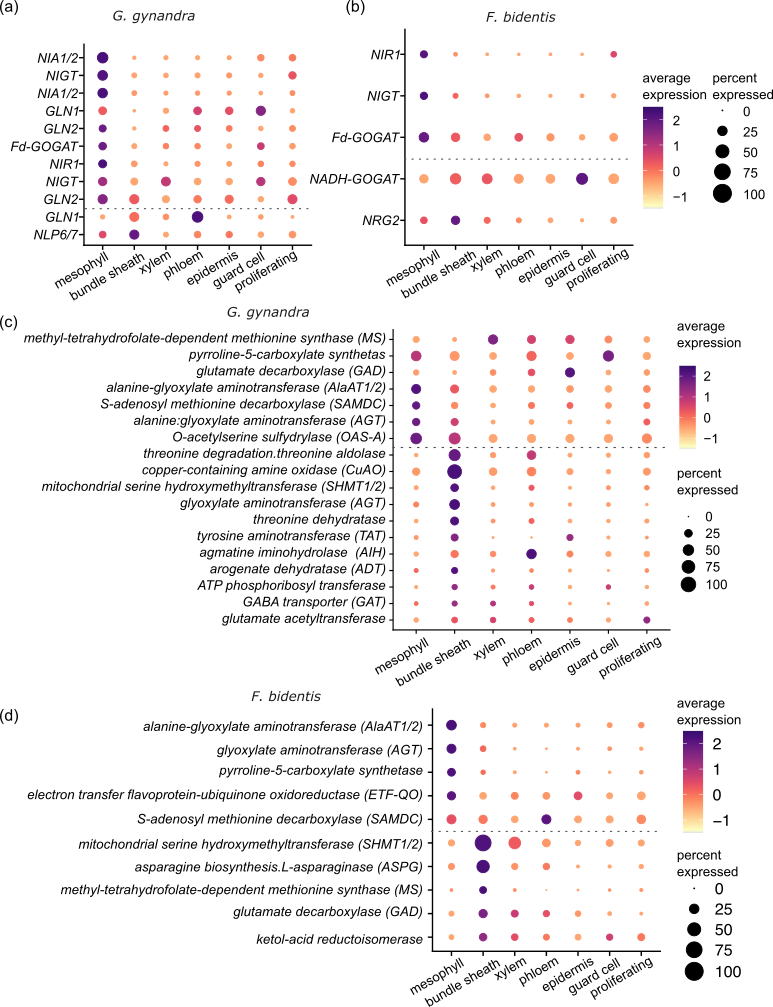


**Fig. S13** Phylogenetic relationship of the species used to identify orthogroups.


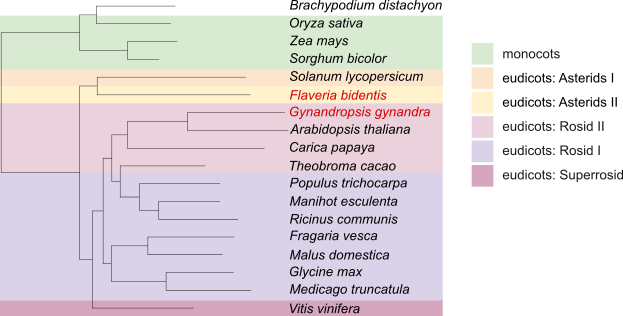


**Fig. S14** Phylogenetic tree of the *PPA* orthogroups. (a) Phylogenetic tree of the *AtPPA1-AtPPA5* orthogroup. (b) Phylogenetic tree of the *AtPPA6* orthogroup. (c) Dot plots showing the expression of *PPA* genes for major cell types. *G. gynandra* and *F. bidentis* genes predicted to be chloroplast-localised are highlighted in the grey box.


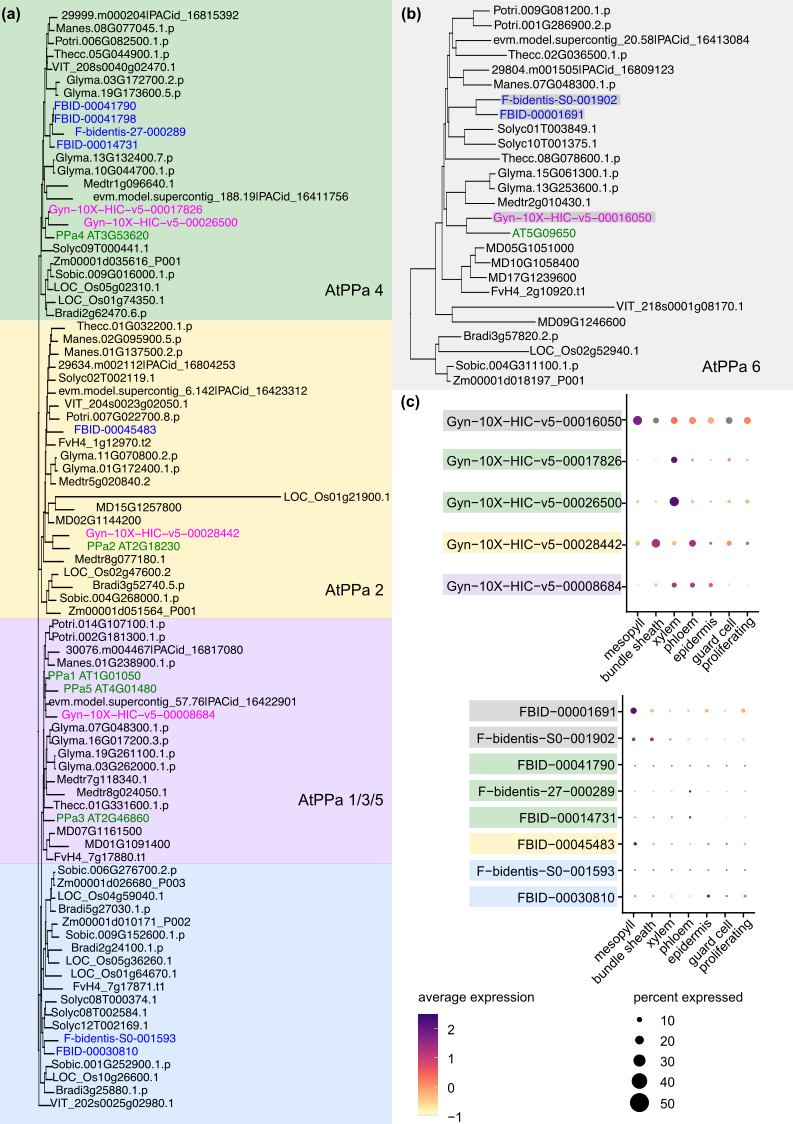


**Fig. S15** Phylogenetic tree of the *PEPC* orthogroup. Expression of individual *PEPC* genes shown in violin plots.


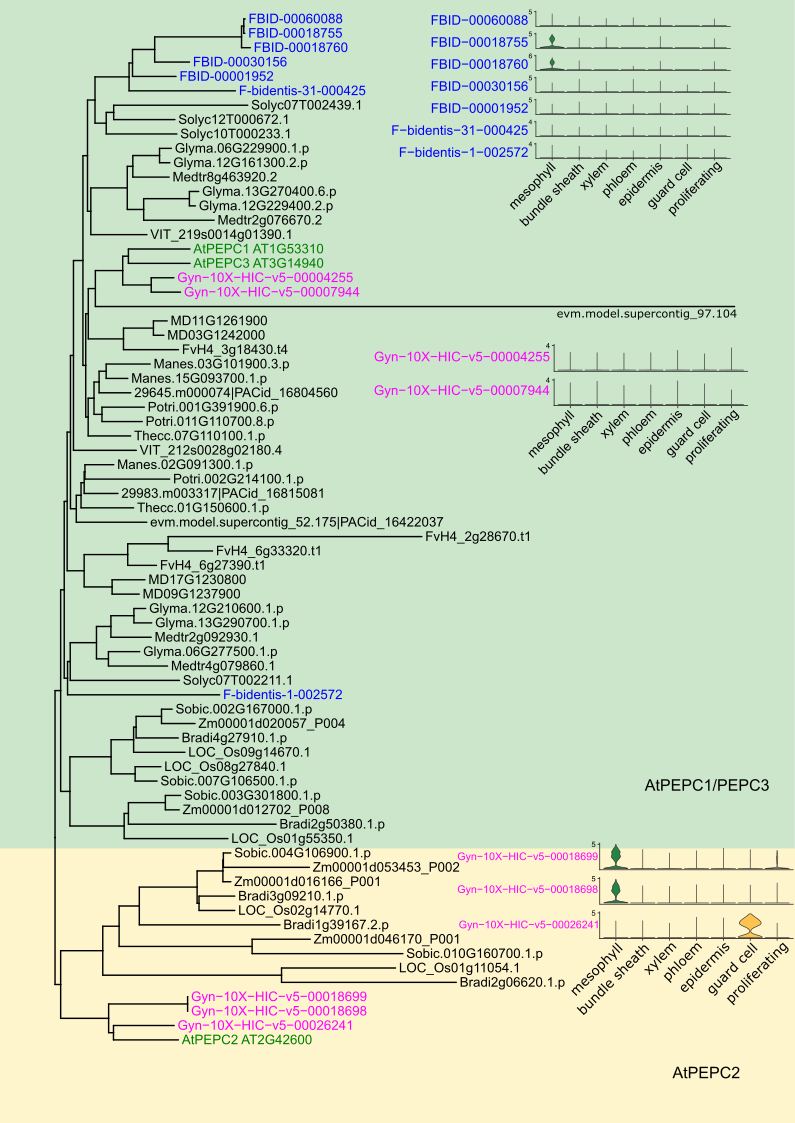


**Fig. S16** Phylogenetic tree of the *AMK* orthogroup. Expression of individual *AMK* genes shown in violin plots.


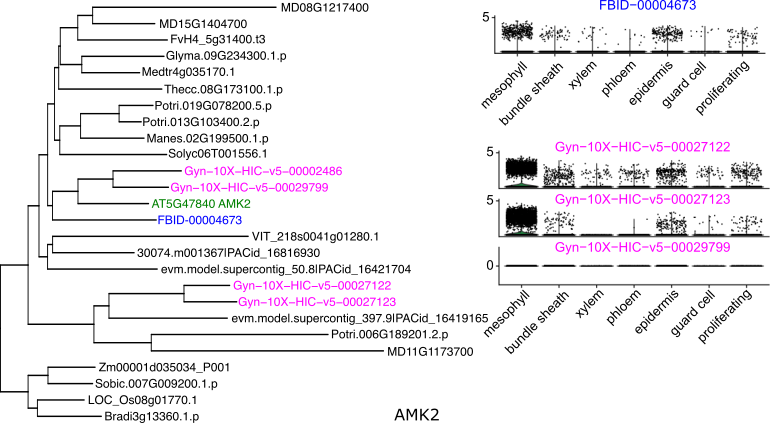


**Fig. S17** Phylogenetic tree of the *CA* orthogroup. Expression of individual *CA* genes shown in violin plots. *G. gynandra* and *F. bidentis* genes predicted to be chloroplast-localised are highlighted in the grey box.

**
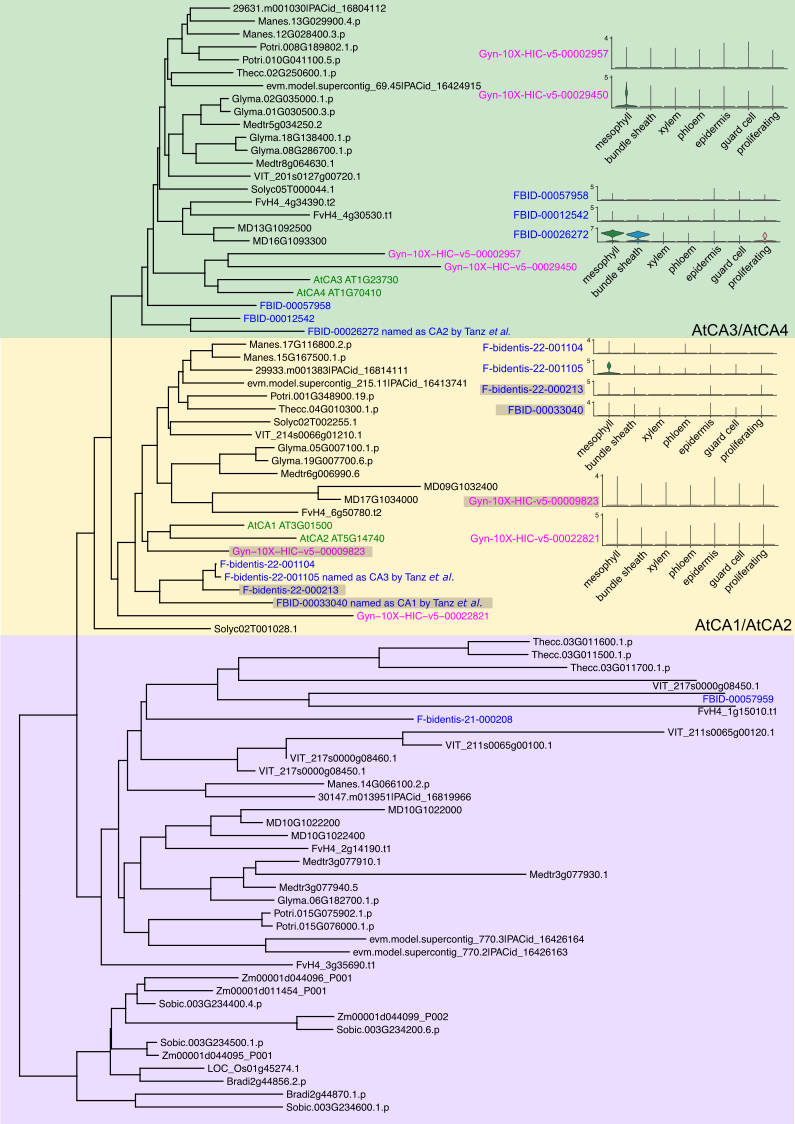
**

**Fig. S18** Expression of differentially expressed genes between mesophyll and bundle sheath. Heatmap of the top 100 genes differentially expressed between mesophyll and bundle sheath cells of *G. gynandra* (a) and *F. bidentis* (b).


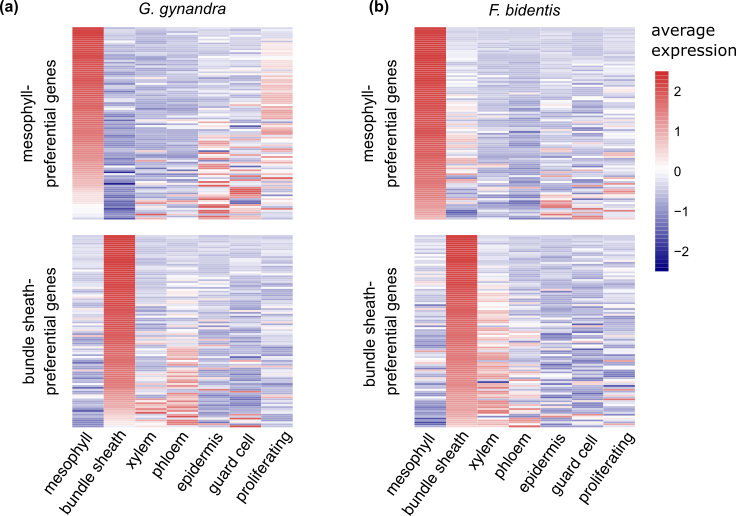


**Fig. S19** Phylogenetic tree of the *NAD-ME* orthogroups. Phylogenetic tree of *AtNAD-ME1* (a) and *AtNAD-ME2* orthogroup (b), expression of individual *NAD-ME* genes shown in violin plots.


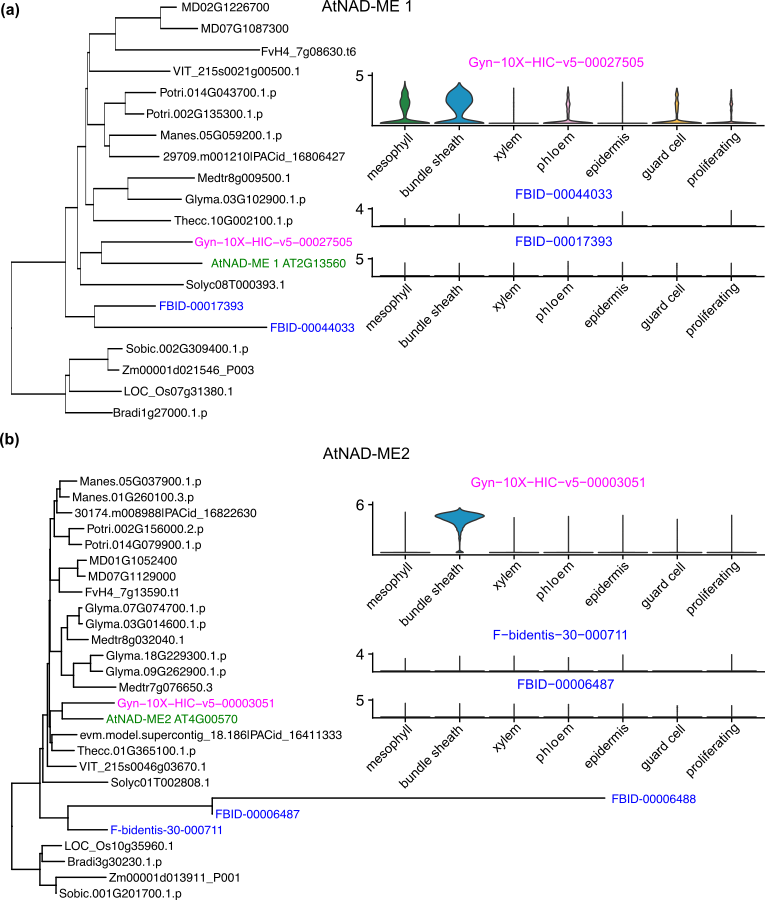


**Fig. S20** Phylogenetic tree of the *NADP-ME* orthogroup. Expression of individual *NADP-ME* genes shown in violin plots.


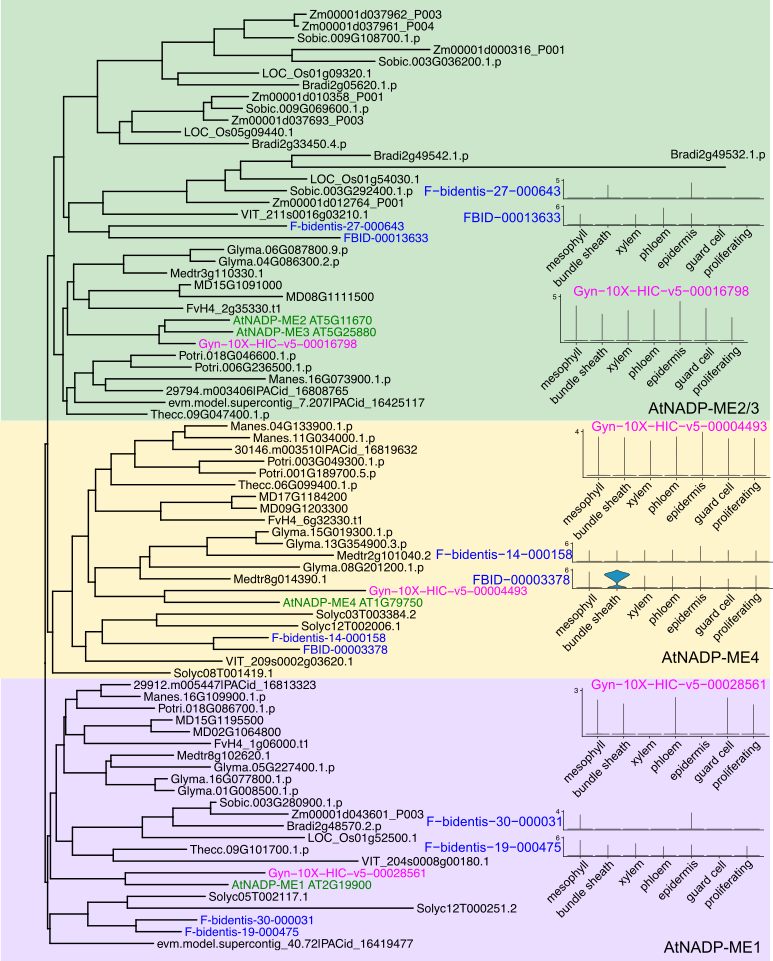


**Fig. S21** Phylogenetic tree of the *PEPCK* orthogroup. Expression of individual *PEPCK* genes shown in violin plots.


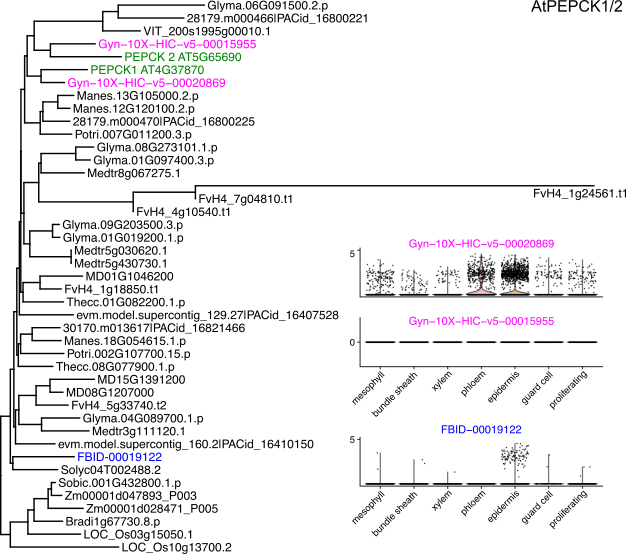


**Fig. S22** Number of all mesophyll and bundle sheath preferential transcription factors characterised into different families in *G. gynandra* and *F. bidentis*.


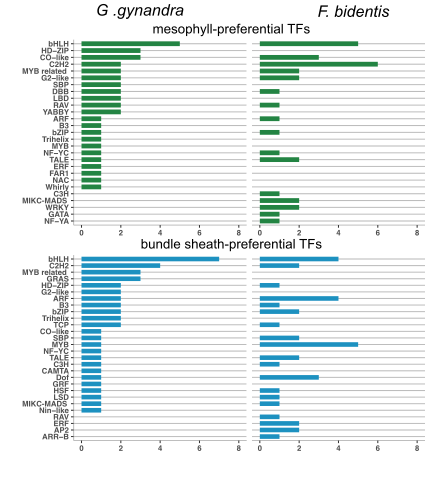


**Fig. S23** Compartmentation of transcription factors between bundle sheath and mesophyll cells using only protoplast data. (a) Number of transcription factors identified in single-nucleus or single-protoplast data of *F. bidentis* and *G. gynandra*. (b) Heatmap of genes differentially expressed between mesophyll and bundle sheath cells. (c) Number of common mesophyll and bundle sheath preferential transcription factors characterised into different families. (d) Transcript abundance of orthologous transcription factors that were preferentially expressed in either mesophyll or bundle sheath cells. Genes identified in both the single-nucleus and protoplast datasets are highlighted in red.


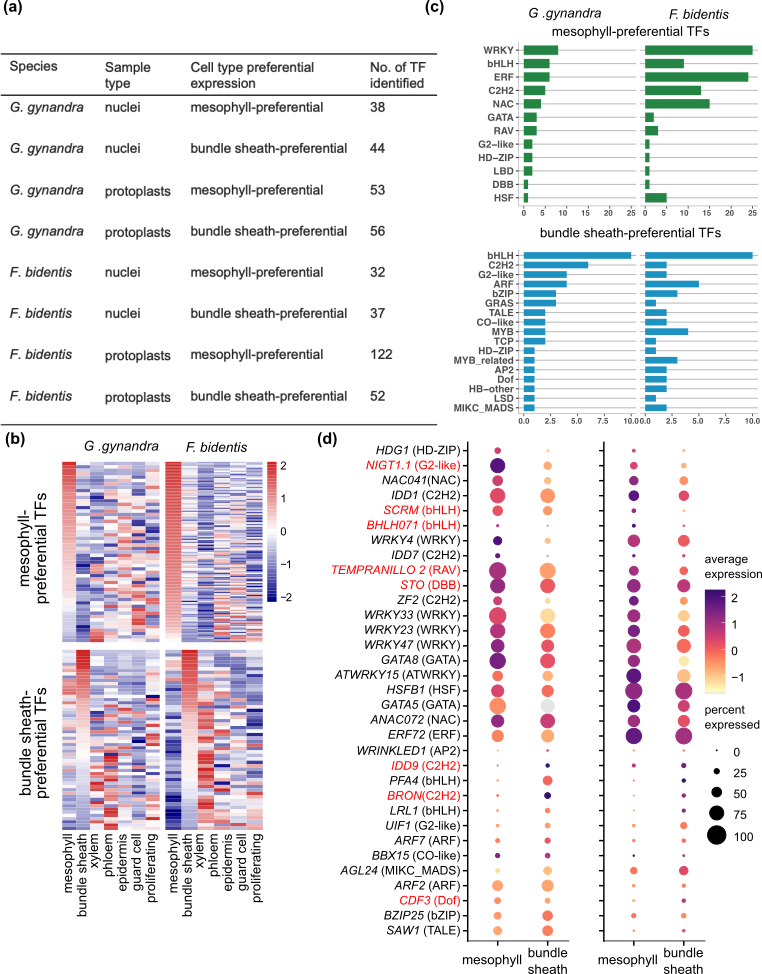


**Fig. S24** UMAP projection of the gene co-expression network and expression patterns of module eigengenes. The topological overlap matrix (TOM) was projected onto a two-dimensional space using UMAP to visualise the structure of the gene co-expression networks of *G. gynandra* (a) and *F. bidentis* (c). Each point represents a gene, coloured by its assigned module; point size corresponds to intramodular connectivity. Clustering patterns reflect the topological similarity of genes within and between modules. The top five most connected transcription factors in each module are labelled, except for Module 5 of *F. bidentis*, where only two transcription factors were identified. Gene expression profiles of each co-expression module are summarised as module eigengenes, and their expression patterns are shown using dot plots for *G. gynandra* (b) and *F. bidentis* (d).


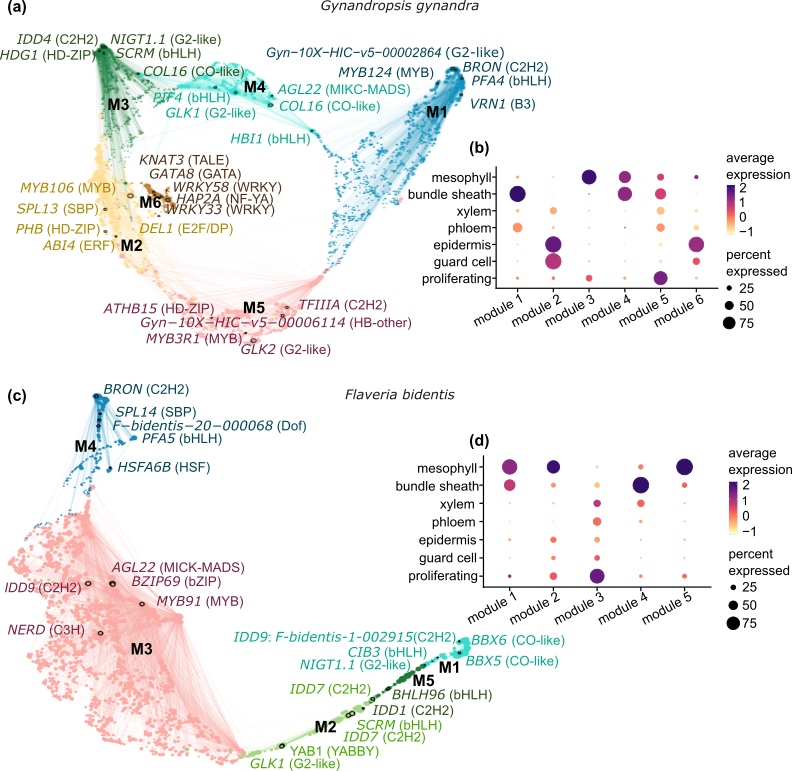

Supplement: Supplementary file 1 — Fig. S1 Workflow illustrating sampling and isolation of nuclei and protoplasts for sequencing. Fig. S2 Representative FACS plots of nuclei sorting. Fig. S3 Optimisation of protoplast isolation for single‐protoplast RNA‐seq. Fig. S4 Clustering and cell type annotation of single‐nucleus and single‐protoplast RNA sequencing from leaves of G. gynandra and F. bidentis. Fig. S5 Cluster annotation for the integrated transcriptome atlases of G. gynandra and F. bidentis leaves. Fig. S6 Number of cell type marker genes shared across different cell types. Fig. S7 Comparison of single‐nucleus and single‐protoplast transcriptome profiles in F. bidentis leaves. Fig. S8 Comparison of gene detection between single‐nucleus and single‐protoplast transcriptome profiles from G. gynandra leaves and cell type representation across replicates. Fig. S9 Comparison of stress and nuclei signature scores. Fig. S10 Expression of photorespiration genes. Fig. S11 Expression of transporter genes. Fig. S12 Expression of nitrogen assimilation and amino acid metabolism genes. Fig. S13 Phylogenetic reconstruction of the species used to identify orthogroups. Fig. S14 Phylogenetic tree of the PPA orthogroups. Fig. S15 Phylogenetic tree of the PEPC orthogroup. Fig. S16 Phylogenetic tree of the AMPK orthogroup. Fig. S17 Phylogenetic tree of the CA orthogroup. Fig. S18 Expression of differentially expressed genes between mesophyll and bundle sheath. Fig. S19 Phylogenetic tree of the NAD‐ME orthogroups. Fig. S20 Phylogenetic tree of the NADP‐ME orthogroup. Fig. S21 Phylogenetic tree of the PEPCK orthogroup. Fig. S22 Number of all mesophyll and bundle sheath‐preferential transcription factors characterised into different families in G. gynandra and F. bidentis. Fig. S23 Compartmentation of transcription factors between bundle sheath and mesophyll using only protoplast data. Fig. S24 UMAP projection of the gene co‐expression network and expression patterns of module eigengenes. [file NPH-249-24-s002.docx]
